# Proceedings of the Canadian Society of Allergy and Clinical Immunology Annual Scientific Meeting 2020

**DOI:** 10.1186/s13223-021-00519-4

**Published:** 2021-03-31

## Allergic Rhinitis/Asthma

### # 01 Hot spots for pediatric asthma emergency department visits in Ottawa, Canada

#### Falana Sheriff^1,2^, Amisha Agarwal^3^, Madhura Thipse^3^, Dhenuka Radhakrishnan^2,3,4^

##### ^1^McMaster University, Hamilton, ON, Canada; ^2^Department of Pediatrics, University of Ottawa, Ottawa, ON, Canada; ^3^Children’s Hospital of Eastern Ontario Research Institute, Ottawa, ON, Canada; ^4^ICES, Toronto, ON, Canada

**Correspondence:** Falana Sheriff

*Allergy Asthma Clin Immunol* 2021, **17(Suppl 1):**01

**Background:** Pediatric asthma emergency department (ED) visits and repeat visits place a significant burden on healthcare. National and provincial level studies demonstrate geographic variation in asthma ED visits and links to marginalization, but preclude translation into practical targeting of healthcare delivery. It is important to understand the relationship between pediatric asthma ED visits and marginalization at a more granular level. Our objective was to map the city-level geographic variation in pediatric asthma ED visit and re-visit rates in Ottawa, Canada and the relationship with marginalization.

**Methods:** We performed a single centre retrospective cohort study of children ages 1–17 with one or more ED visits for asthma at the Children’s Hospital of Eastern Ontario. Using postal codes, we linked patients to census tracts. Per census tract, we mapped pediatric asthma ED visit and re-visit rates within 1 year and identified overlap with the Ontario Marginalization Index.

**Results:** Of 1,620 children with an index ED visit, 18.5% had a repeat ED visit. We identified 10 hot spot census tracts each for pediatric asthma ED visit and re-visit rates. We identified an overlap between urban hot spots and areas with high marginalization with respect to residential instability, material deprivation and ethnic concentration.

**Conclusions:** At a granular, city-wide level, pediatric asthma ED visit and re-visit rates are heterogeneous. Urban hot spots, in contrast to rural, have more overlap with marginalization. These methods can be used in other jurisdictions to inform practical community strategies for geographically-targeted prevention of pediatric asthma-related ED visits in vulnerable areas.

### #02 A mild case of COVID-19 infection in a 69-year old male with severe eosinophilic asthma on mepolizumab

#### Hoang Pham^1^, Derek Lanoue^2^, Jodi Valois^3^, Tim Olynych^2, 3^, William Yang^3,4^

##### ^1^Division of Clinical Immunology and Allergy, Department of Medicine, McGill University Health Centre, Montreal, QC, Canada; ^2^Department of Medicine, The Ottawa Hospital, The University of Ottawa, Ottawa, ON, Canada; ^3^Ottawa Allergy Research Corporation, Ottawa, ON, Canada; ^4^Faculty of Medicine, University of Ottawa, Ottawa, ON, Canada

**Correspondence:** Jodi Valois

*Allergy Asthma Clin Immunol* 2021, **17(Suppl 1):**02

**Background:** Since the emergence of COVID-19, clinicians have struggled to predict which patients will have more severe clinical courses. The CDC has listed moderate-severe asthma as a possible risk factor for severe COVID-19 infection. Type 2 targeted biologic treatments are recommended as add-on therapy for moderate-severe asthmatics with uncontrolled symptoms. We hypothesize that achieving good control of chronic asthma through the use of these agents will allow patients to have improved outcomes. As such, we describe a reassuring case of COVID-19 in a severe eosinophilic asthmatic on mepolizumab.

**Case Presentation:** We present a 69-year old Caucasian male with late-onset eosinophilic non-atopic asthma controlled on mepolizumab with a baseline FEV1 of 96% and absolute eosinophil count of 1300 cells/μmol. His medical history was significant for obesity (BMI 31) and remote 14-pack year smoking history but no cardiac comorbidities. On March 29th, 2020, he tested positive for COVID-19 after 2 days of worsening symptoms with known exposure. His presenting symptoms consisted of a dry cough and headache for 3 days which progressed to profound fatigue followed by arthralgias and myalgias. His monthly mepolizumab was withheld and he was temporized with high dose mometasone/formoterol and tiotropium inhalers. He recovered completely at home within one-week without needing prednisone, antibiotics, emergency department, or hospitalization. Mepolizumab was restarted on May 27, 2020.

**Conclusions:** Despite advanced age, obesity, and severe eosinophilic asthma, our patient had only a mild infection with COVID-19. This case highlights the importance of achieving optimal asthma control with the established array of asthma therapies, including biologic targeted treatments against IL-5, to prevent a severe exacerbation with this novel respiratory infection. Dedicated cohort and mechanistic studies with well characterized asthmatics with COVID-19 are needed to more clearly understand risk and severity outcomes as well as to clarify guidance on withholding or continuing biologics in asthmatics infected with COVID-19.

**Statement of Consent:** Written informed consent for this case report was obtained from the patient.

### #03 Investigating autoantibody-mediated macrophage dysfunction in severe asthmatics with recurrent infections

#### Kate H. Miyasaki^1^, Kiho Son^1^, Nan Zhao^1^, Katherine Radford^2^, Chynna Huang^2^, Nicola LaVigne^2^, Joseph Chon^1^, Dessi Loukov^1^, Dawn Bowdish^1^, Parameswaran Nair^1, 2^, Manali Mukherjee^1,2^

##### ^1^McMaster University, Hamilton, ON, Canada; ^2^St. Joseph’s Healthcare Hamilton, Hamilton, ON, Canada

**Correspondence:** Kate H. Miyasaki

*Allergy Asthma Clin Immunol* 2021, **17(Suppl 1):**03

**Background:** Increased levels of sputum autoantibodies (aAbs) were reported in the airways of severe eosinophilic asthmatics with recurrent infective bronchitis, referred to as a mixed phenotype (Mukherjee et al. JACI 2018). We hypothesized the presence of autoantibodies against macrophage scavenger receptors affect monocyte and macrophage response to infection and contribute to infection susceptibility.

**Methods:** Sputum anti-eosinophil peroxidase (EPX) IgG and anti-macrophage receptor with collagenous structure (MARCO) titers were determined by in-house developed ELISA. Adherent mononuclear cells (predominant monocyte population) were isolated from peripheral blood of n = 4 healthy volunteers and used directly or differentiated into monocyte-derived macrophages (MDMs). MDMs and monocytes (at 2 × 10^5^ cells/well) were incubated with 2 µg eluted immunoprecipitated immunoglobulins (IP-Igs) from sputa with high anti-MARCO titers (n = 10) or non-specific IgG from healthy donors (ChromPure, Jackson ImmunoResearch) for 48 h at 37 °C ± 100 ng/mL lipopolysaccharide (LPS) and assessed for cytokine release (Eve Technologies, Alberta).

**Results:** Anti-MARCO IgG was detected at high titers (up to 1:16) in n = 81 eosinophilic asthmatics(EA) with recurrent infections (mixed granulocytic sputa), compared to n = 47 prednisone-dependent EAs, n = 42 ICS-dependent EAs, n = 16 neutrophilic, n = 7 paucigranulocytic asthmatics and n = 16 healthy volunteer sputa (P < 0.05, 2-way ANOVA). The anti-MARCO IgG positively correlated with anti-EPX IgG (R = 0.87, P < 0.0001) and markers of airway infection, sputum total cell count (r = 0.32, p < 0.0001) and absolute sputum neutrophils (r = 0.23, p = 0.001). Monocytes and autologous MDMs incubated with IP-Ig with high sputum aAb titers showed a significant decrease in IL-6, IL-10 and granulocyte macrophage-colony stimulating factor, while monocytes showed reduced interferon-gamma and IL-1b release compared to non-specific IgG controls (p < 0.05, 2-way ANOVA, Tukey’s correction) in response to LPS.

**Conclusions:** We report that presence of sputum aAbs against Mφ proteins, in particular scavenger receptors, could impede effective host defense and lead to recurrent infective bronchitis in eosinophilic asthmatics.

### #04 Clinical validation of the environmental exposure unit for house dust mite exposure

#### Lubnaa Hossenbaccus^1^, Lisa M. Steacy^2^, Terry Walker^2^, Crystal Malone^2^, Anne K. Ellis^1,2^

##### ^1^Queen’s University, Kingston, ON, Canada; ^2^KHSC, Kingston General Hospital site, Kingston, ON, Canada

**Correspondence:** Lubnaa Hossenbaccus

*Allergy Asthma Clin Immunol* 2021, **17(Suppl 1):**04

**Background:** The Environmental Exposure Unit (EEU) is a controlled allergen exposure facility used to study allergic rhinitis (AR). It has been previously evaluated for use with seasonal allergens including ragweed, birch, and grass pollens. To study the perennial allergen, house dust mite (HDM), a dedicated room was constructed within the EEU, termed the HDM-EEU. This study serves to clinically validate the HDM-EEU for HDM-induced AR.

**Methods:** Eligible HDM-allergic participants were invited to a 3-h HDM exposure session in the HDM-EEU following confirmation of relevant clinical history and positive skin prick tests to *Dermatophagoides pteronyssinus* (*D. pteronyssinus*) and *Dermatophagoides farinae* (*D. farinae*). Participants were trained to collect AR data, consisting of Total Nasal Symptom Score (TNSS) and Peak Nasal Inspiratory Flow (PNIF). TNSS and PNIF were recorded at baseline, every half an hour while in the HDM-EEU, and on an hourly basis for up to 24 h following the termination of allergen exposure. Blood samples for biological analyses were collected pre- and post-allergen exposure.

**Results:** A total of 44 allergic and 11 non-allergic participants were included in this study. Compared to controls, HDM-allergics had significantly elevated TNSS (p < 0.05) for up to 6 h post-exposure and significantly decreased % PNIF change (p < 0.05) from baseline at hours 2 and 3 while in the HDM-EEU. Strong correlations observed between TNSS vs PNIF (R^2^ = 0.9254) and PNIF vs nasal congestion (R^2^ = 0.9388) suggest that participants were effectively trained to report their symptoms accurately. Serum analyses revealed significantly elevated concentrations of specific immunoglobulin E (sIgE) against *Der p 1* and *Der f 1* (p < 0.0001) in HDM-allergics than non-allergics, with a strong correlation in sensitization to both allergens (R^2^ = 0.9439).

**Conclusions:** The HDM-EEU is a suitable clinical model to induce AR symptoms in HDM-sensitized participants to investigate its symptomatic and biological manifestations.

### #05 *Staphylococcus aureus* carriage and biological responses of ragweed pollen-induced allergic rhinitis using the nasal allergen challenge model

#### Sophia Linton^1,2^, Jenny Thiele^1,2^, Lisa M. Steacy^2^, Prameet M. Sheth^3,4,5^, Anne K. Ellis^1,2^

##### ^1^Department of Medicine, Queen’s University, Kingston, ON, Canada; ^2^Allergy Research Unit, Kingston Health Sciences Centre - KGH Site, Kingston, ON, Canada; ^3^Department of Pathology and Molecular Sciences, Queen’s University, Kingston, ON, Canada; ^4^Division of Microbiology, Kingston Health Sciences Centre - KGH Site, Kingston, ON, Canada; ^5^Gastrointestinal Disease Research Unit, Kingston Health Sciences Centre - KGH Site, Kingston, ON, Canada

**Correspondence:** Sophia Linton

*Allergy Asthma Clin Immunol* 2021, **17(Suppl 1):**05

**Background:** Little is known about the impact of the nasal microbiome on allergic rhinitis (AR). The current study aimed to determine if Ragweed (RG)-allergics were more likely to be colonized with *Staphylococcus aureus* in the anterior nares using the nasal allergen challenge model (NAC).

**Methods:** Consenting individuals (20 RG-allergic and 11 non-allergic) were screened for *S. aureus* colonization with a nasal swab before receiving increasing concentrations of ragweed pollen extract intranasally until each participant achieved qualifying criteria for a positive NAC (Total Nasal Symptom Score(TNSS) ≥ 8 and %Peak Nasal Inspiratory Flow(PNIF) fall ≥ 50%). 15 RG-allergic and 9 non-allergic participants qualified for NAC. Peripheral blood samples were collected at baseline, 1 h, 6 h, and 24 h post-NAC. RG-specific IgE (sIgE) was measured using ImmunoCAP assays from the collected serum. All statistical analyses were performed using GraphPad Prism 7.0.

**Results:** Seven consenting individuals were colonized with methicillin-sensitive *S. aureus*. 85.7% of *S. aureus* carriers were RG-allergic (n = 6), and 14.3% were non-allergic (n = 1). Peripheral blood eosinophil levels were significantly decreased in RG-allergics 1 h and 6 h post-NAC compared to baseline (both, p < 0.05). There were no significant differences in white blood cell counts between RG-allergics and non-allergics at baseline, 1 h, 6 h, or 24 h post-NAC. RG-sIgE levels were significantly higher in RG-allergics than non-allergics (p > 0.0001). RG-allergics colonized with *S. aureus* had significantly greater blood leukocyte levels at 6H than RG-allergics without *S. aureus* (p = 0.0234). There was no correlation between RG-sIgE levels and *S. aureus* carriage in RG-allergics (Spearman r = − 0.05270, p = 0.8788).

**Conclusions:** More RG-allergics were colonized with *S. aureus* in the anterior nares than non-allergics, though not significantly. In this study, there was no correlation between *S. aureus* carriage and biological responses of ragweed pollen-induced AR.

### #06 Efficacy of dupilumab in chronic rhinosinusitis with nasal polyps and comorbid asthma by baseline biomarkers of type 2 inflammation from the pooled population of the SINUS-24 and SINUS-52 phase 3 trials

#### Jorge F. Maspero^1^, Claus Bachert^2,3^, G. Walter Canonica^4^, Brian N. Swanson^5^, Seong H. Cho^6^, Sivan Harel^7^, Alexandre Jagerschmidt^8^, Mei Zhang^9^, Xuezhou Mao^9^, Leda P. Mannent^8^, Nikhil Amin^7^

##### ^1^Fundación CIDEA, Buenos Aires, Argentina; ^2^Ghent University, Ghent, Belgium; ^3^Karolinska Institutet, Stockholm, Sweden; ^4^Humanitas University and Research Hospital, Milan, Italy; ^5^ Thomas Jefferson University, Philadelphia, PA, USA; ^6^University of South Florida, Tampa, FL, USA; ^7^Regeneron Pharmaceuticals, Inc., Tarrytown, NY, USA; ^8^Sanofi, Chilly-Mazarin, France; ^9^Sanofi, Bridgewater, NJ, USA

**Correspondence:** Jorge F. Maspero

*Allergy Asthma Clin Immunol* 2021, **17(Suppl 1):**06

**Background:** Chronic rhinosinusitis with nasal polyps (CRSwNP) and asthma share type 2 inflammatory pathophysiology and are frequent comorbidities. Dupilumab, a fully human VelocImmune^®^-derived monoclonal antibody, blocks the shared receptor component for IL-4 and IL-13, key and central drivers of type 2 inflammation and regulators of IgE, thymus and activation-regulated chemokine (TARC), and eotaxin-3 expression. We report the effect of dupilumab on upper and lower airway outcomes in patients with CRSwNP and comorbid asthma from the SINUS-24/52 studies (NCT02912468/NCT02898454), categorized by baseline IgE, TARC, and eotaxin-3.

**Methods:** Patients were randomized to subcutaneous dupilumab 300 mg or placebo every 2 weeks for 24 weeks. Dupilumab treatment effects were evaluated in patients with asthma, grouped by ≥/< baseline median serum total IgE (120 IU/mL), TARC (291 pg/mL), and eotaxin-3 (60.5 pg/mL).

**Results:** For patients with asthma (428/724, 59.1%) mean (SD) baseline IgE, TARC, and eotaxin-3 were 249.12 (308.82) IU/mL, 377.44 (266.13) pg/mL, and 81.02 (78.36) pg/mL, respectively. Patients had severe upper/lower airway disease at baseline (mean [SD] for: nasal polyp score [NPS] 5.97 [1.26], range 0–6; nasal congestion [NC] score 2.42 [0.57], range 0–3; nasal peak inspiratory flow [NPIF] 87.02 [57.26] L/minute; and 6-item Asthma Control Questionnaire [ACQ-6] score 1.59 [1.10], range 0–6). At Week 24, all outcomes were improved (*P* < 0.01) with dupilumab vs placebo, regardless of baseline IgE/TARC/eotaxin-3 levels (LS mean change ≥/< 120 IU/mL IgE, 291 pg/mL TARC, and 60.5 pg/mL eotaxin-3, respectively, for: NPS, −2.18/−1.88, −2.20/−1.83, and −2.30/−1.73; NC score, −1.07/−1.02, −1.10/−0.98, and -−1.17/−0.88; NPIF, 51.96/38.59, 54.38/35.91, and 55.85/32.89; and ACQ-6 score, −0.79/−0.87, −0.83/−0.78, and −0.91/−0.66).

**Conclusions:** Patients with CRSwNP and comorbid asthma had severe upper and lower airway disease at baseline. Dupilumab improved upper and lower airway outcome measures in these patients regardless of baseline IgE, TARC, or eotaxin-3.

### #07 Treatment effect of the tree pollen SLIT-tablet on allergic rhinoconjunctivitis during oak pollen season

#### Hendrik Nolte^1^, Susan Waserman^2^, Anne K. Ellis^3^, Peter A. Würtzen^4^, Tilo Biedermann^5^

##### ^1^ALK, Bedminster, NJ, USA; ^2^Department of Medicine, McMaster University, Hamilton, ON, Canada; ^3^Department of Medicine, Queen’s University, Kingston, ON, Canada; ^4^ALK, Hørsholm, Denmark; ^5^Department of Dermatology and Allergology, Technical University of Munich, Munich, Germany

**Correspondence:** Hendrik Nolte

*Allergy Asthma Clin Immunol* 2021, **17(Suppl 1):**07

**Background:** The birch homologous group contains several tree-species, including oak, based on IgE cross-reactivity to the birch allergens Bet v 1 and Bet v 2. In this post hoc analysis, treatment effects of the tree pollen sublingual immunotherapy (SLIT)-tablet containing standardized birch pollen extract in participants with birch and related pollen-induced allergic rhinitis with or without conjunctivitis (AR/C) were evaluated during a birch-free oak pollen season.

**Methods:** In a randomized, multinational, double-blind trial (EudraCT-2015-004821-15), 634 participants (12-65 years) received daily tree SLIT-tablet (12 SQ-Bet) or placebo before and during tree pollen season (February-May). Symptom-relieving medication use was allowed. The total combined rhinoconjunctivitis symptom and medication score (TCS), the rhinoconjunctivitis daily symptom score (DSS), the rhinoconjunctivitis daily medication score (DMS), and immunologic parameters were analyzed post hoc during the oak pollen season, which excluded any data from days overlapping the birch pollen season.

**Results:** Only 1% of participants were IgE mono-sensitized to birch pollen while 87% were also sensitized to oak pollen. Relative improvements in TCS, DSS, and DMS with the tree SLIT-tablet versus placebo during the oak pollen season were 25%, 22%, and 32% (all p < 0.001). Significant correlations were observed between birch and oak pollen sIgE at baseline (r = 0.86) and between birch and oak pollen IgG_4_ after treatment (r = 0.72). Oak pollen sIgE and IgG_4_ immune responses to tree SLIT-tablet treatment were similar to birch pollen.

**Conclusions:** Improvements with tree SLIT-tablet versus placebo during the oak pollen season support the clinical relevance of the immunologic cross-reactivity between birch and oak pollen. The tree SLIT-tablet could be used to treat oak pollen-related AR/C symptoms and reduce the need for symptom-relieving pharmacotherapy.

### #08 Evaluation of sublingual immunotherapy (SLIT) class effects across the tree SLIT-tablet development program

#### Marie Chantal Arseneault^1^, Lars Frølund^2^, Mandy Cuevas^3^, Morgan Andersson^4^, Ida M. Smith^5^, Tina M. Greve^5^

##### ^1^ALK Canada, Montreal, QC, Canada; ^2^Allergy and Lung Clinic, Helsingør, Denmark; ^3^Department of Allergology/Rhinology, Clinic for Otorhinolaryngology and Head and Neck surgery, Carl-Gustav Carus University Clinic Dresden, Dresden, Germany; ^4^Department of Otorhinolaryngology, Lund, University Hospital Skane, Lund, Sweden; ^5^ALK, Hørsholm, Denmark

**Correspondence:** Marie Chantal Arseneault

*Allergy Asthma Clin Immunol* 2021, **17(Suppl 1):**08

**Background:** The tree sublingual (SLIT)-tablet has been developed for treatment of allergic rhinoconjunctivitis induced by pollen from the birch homologous group. Risks identified as class effects of SLIT were analyzed across the clinical development program.

**Methods:** Safety data from 2 phase II trials and 1 phase III trial were pooled (12 SQ-Bet, N = 471; placebo, N = 458) and analyzed for severe events within the following areas: Laryngo-pharyngeal reactions (38 preferred terms [PTs]), systemic allergic reactions (MedDRA SMQ Anaphylactic reaction), acute asthma worsening, and eosinophilic esophagitis (EoE).

**Results:** Treatment-related laryngo-pharyngeal reactions were more frequent with 12 SQ-Bet (54% of subjects) than placebo (11%). The most commonly reported PT was throat irritation (29% of 12 SQ-Bet subjects; 4% placebo). Most reactions were mild or moderate in intensity, occurred early during treatment, and were transient. Nine severe swellings in the oral cavity or throat were reported by eight 12 SQ-Bet subjects and one placebo subject. No laryngo-pharyngeal reactions were serious or compromised the airways.

No cases of anaphylactic reaction were reported in 12 SQ-Bet subjects. Most potential systemic allergic reactions identified by the SMQ involved mild or moderate symptoms and led to no change in treatment; all were manageable and resolved either spontaneously or with conventional pharmacotherapy. No adrenaline use was reported.

Asthma events were reported by seven 12 SQ-Bet subjects and ten placebo subjects; most events were mild or moderate and unrelated to treatment. Overall, the pooled data suggest no increased risk of acute asthma worsening associated with 12 SQ-Bet.

No events of EoE were reported.

**Conclusions:** The safety profile of the tree SLIT-tablet is consistent with that of other SLIT-tablets. Laryngo-pharyngeal reactions were frequent but manageable and none resulted in airway obstruction. The data indicated no increased risk of acute asthma worsening or anaphylactic reactions, and no cases of EoE were reported.

### #09 Dupilumab reduced severe exacerbations and improved lung function across baseline IgE levels in patients with elevated baseline blood eosinophils in LIBERTY ASTHMA QUEST

#### Warner W. Carr^1^, Jonathan Corren^2^, Thomas B. Casale^3^, Nadia Daizadeh^4^, Asif H. Khan^5^, Siddhesh Kamat^6^, Yamo Deniz^6^, Paul Rowe^7^

##### ^1^Allergy & Asthma Associates of Southern California, Mission Viejo, CA, USA; ^2^David Geffen School of Medicine at UCLA, Los Angeles, CA, USA; ^3^University of South Florida, Tampa, FL, USA; ^4^Sanofi, Cambridge, MA, USA; ^5^Sanofi, Chilly Mazarin, France; ^6^Regeneron Pharmaceuticals Inc., Tarrytown, NY, USA; ^7^Sanofi, Bridgewater, NJ, USA

**Correspondence:** Warner W. Carr

*Allergy Asthma Clin Immunol* 2021, **17(Suppl 1):**09

**Background:** Dupilumab, a fully human VelocImmune^®^-derived monoclonal antibody, blocks the shared receptor component for IL-4 and IL-13, key and central drivers of type 2 inflammation in multiple diseases. In phase 3 LIBERTY ASTHMA QUEST (NCT02414854), add-on dupilumab 200 mg or 300 mg every 2 weeks (q2w) vs placebo significantly reduced severe asthma exacerbation rates and improved forced expiratory volume in 1 s (FEV_1_) in patients with uncontrolled, moderate-to-severe asthma. Treatment effects were greater in patients with elevated baseline type 2 biomarkers. A large proportion of asthma patients with type 2 inflammation also have allergic asthma, including IgE-mediated asthma. This post hoc analysis assessed dupilumab effects in patients with baseline blood eosinophils ≥ 150μL categorized by baseline total serum IgE levels (< 100; 100 to < 500; ≥ 500 IU/mL).

**Methods:** Annualized severe exacerbation rates during the 52-week treatment period were analyzed using negative binomial regression models. The least squares mean change from baseline in pre-bronchodilator FEV_1_ at Weeks 12 and 52 was analyzed using a mixed-effect model with repeated measures.

**Results:** Of 1902 patients enrolled in QUEST, 71% had baseline eosinophils ≥ 150μL. In this group, baseline characteristics were generally comparable between the dupilumab and placebo groups across baseline IgE levels. Dupilumab 200 mg and 300 mg q2w combined vs placebo significantly reduced severe exacerbation rates by 59%, 53%, and 65% in the groups with baseline IgE level < 100, 100 to < 500, and ≥ 500 IU/mL, respectively (all *P *< 0.0001). Dupilumab vs placebo also improved FEV_1_ at Week 12 by 0.07L (*P *= 0.07), 0.18L, and 0.21L, and at Week 52 by 0.14L, 0.21L, and 0.26L in the groups with baseline IgE levels < 100, 100 to < 500, and ≥ 500 IU/mL, respectively (all *P *< 0.001 unless otherwise stated).

**Conclusions:** Dupilumab significantly reduced severe asthma exacerbations and improved pre-bronchodilator FEV_1_ across all IgE subgroups in patients with uncontrolled, moderate-to-severe asthma and with baseline blood eosinophils ≥ 150μL.

### #10 Dupilumab reduced oral corticosteroid (OCS) use in patients with OCS-dependent, severe asthma across baseline disease characteristics: LIBERTY ASTHMA VENTURE Study

#### Mario Castro^1^, Nicola A. Hanania^2^, Nadia Daizadeh^3^, Benjamin Ortiz^4^, Nami Pandit-Abid^5^, Yamo Deniz^4^, Paul Rowe^5^

##### ^1^University of Kansas School of Medicine, Kansas City, KS, USA; ^2^Baylor College of Medicine, Houston, TX, USA; ^3^Sanofi, Cambridge, MA, USA; ^4^Regeneron Pharmaceuticals, Inc., Tarrytown, NY, USA; ^5^Sanofi, Bridgewater, NJ, USA

**Correspondence:** Mario Castro

*Allergy Asthma Clin Immunol* 2021, **17(Suppl 1):**10

**Background:** Dupilumab, a fully human VelocImmune^®^-derived monoclonal antibody, blocks the shared receptor component for IL-4/IL-13, key and central drivers of type 2 inflammation in multiple diseases. In phase 3 VENTURE (NCT02528214), add-on dupilumab 300 mg every 2 weeks vs placebo reduced OCS use and severe asthma exacerbations, and improved pre-bronchodilator FEV_1_ in patients with OCS-dependent, severe asthma. This post hoc analysis assessed dupilumab effects on OCS use by baseline disease characteristics.

**Methods:** Percentage reductions in OCS dose (mg/day) at Weeks 12 (Wk12) and 24 (Wk24) were compared between dupilumab- and placebo-treated patients grouped by baseline characteristics.

**Results:** Least squares mean differences (95% CI) in OCS dose percentage reduction from baseline between dupilumab and placebo were (all *P* values for interaction> 0.05): FEV_1_ ≤ 1.75 and > 1.75 L, Wk12, 14.77% (0.94, 28.61) and 25.31% (8.24, 42.38), respectively; Wk24, 31.68% (15.88, 47.48) and 32.21% (11.15, 53.26); FEV_1_ < 60% and ≥ 60%, Wk12, 19.67% (5.82, 33.52) and 13.06% (− 0.25, 26.36); Wk24, 32.44% (17.14, 47.74) and 25.06% (5.87, 44.26); 5-item Asthma Control Questionnaire score ≤ 2 and > 2, Wk12, 17.87% (0.98, 34.76) and 14.86% (1.30, 28.42); Wk24, 35.84% (16.05, 55.63) and 23.95% (7.80, 40.10); former/never smokers, Wk12, 12.09% (− 18.34, 42.52) and 18.15% (6.83, 29.46); Wk24, 21.31% (− 5.95, 48.58) and 30.65% (17.00, 44.29); with/without an ongoing atopic medical condition, Wk12, 16.97% (5.22, 28.72) and 14.11% (− 9.59, 37.81); Wk24, 30.95% (17.14, 44.76) and 20.78% (− 7.03, 48.59); onset age < 18, 18–40, and > 40 years, Wk12, 10.72% (− 13.05, 34.50), 19.19% (2.46, 35.92), and 16.65% (− 2.31, 35.61); Wk24, 29.20% (2.60, 55.79), 32.02% (9.80, 54.23), and 20.88% (2.87, 38.90); ≤ 1 and > 1 previous year severe exacerbation, Wk12, 22.65% (8.70, 36.60) and 8.71% (− 6.63, 24.05); Wk24, 25.11% (9.07, 41.15) and 28.21% (9.74, 46.68).

**Conclusions:** Dupilumab reduced OCS use across all baseline disease characteristics in patients with OCS-dependent, severe asthma. No significant treatment-by-subgroup interactions were identified.

## Allied Health

### #11 Nurse led development of an atopic dermatitis education program

#### Shauna Filuk^1,2^, Jo-Anne St. Vincent^1,2^, Shannon Deane^1,2^, Diane Marks^1,2^, Allan Becker^1,2^, Elinor Simons^1,2^

##### ^1^The Children’s Allergy & Asthma Education Centre (CAAEC), Winnipeg, MB, Canada; ^2^Section of Allergy & Clinical Immunology, Department of Pediatrics & Child Health, Children’s Hospital of Manitoba, Winnipeg, MB, Canada

**Correspondence:** Shauna Filuk

*Allergy Asthma Clin Immunol* 2021, **17(Suppl 1):**11

**Background:** Atopic dermatitis (AD) is a chronically relapsing inflammatory skin disease associated with significant impairment of the caregiver’s and child’s quality of life. The treatment is not curative and daily maintenance therapy is required. Our purpose was to develop and evaluate an education program to help parents of young children gain knowledge and skills to successfully manage AD over time.

**Methods:** Program development included a literature review and scan of AD education programs available across Canada and North America. We collaborated with experts in the field, including National Jewish Health Center’s Eczema Treatment Program, and with local allergists, dermatologists and parents of children with severe AD.

The standardized AD Education Program we developed was directed at parents of children under age 5 years with moderate-to-severe AD (SCORAD > 25) and included a knowledge portion and directed demonstration and practice of emollient application, prescribed medications and specialized interventions such as wet wraps.

A parent satisfaction questionnaire was sent to families post AD education via Survey Monkey™. Program information was sent to all pediatricians in Winnipeg and advertised on our website (www.caaec.ca).

**Results:** Of the 187 children referred to the program, 63 (34%) received education, 114 (61%) did not respond to multiple invitations and 10 (5%) declined education. Of the families who received education, 30 children (48%) had severe, 15 (24%) had moderate AD and SCORAD was unavailable for 18 (28%). Families reported a high level of program satisfaction with a sense of improved knowledge, increased skills, awareness, and benefits for their child.

**Conclusions:** AD education programs are rare but help to enhance children’s AD management. Information, demonstration and support provided to families can improve overall satisfaction, compliance and treatment efficacy for AD. Offering the education program to more families will give the CAAEC further feedback and evaluation on its effectiveness.

## Food Allergy/Anaphylaxis

### #12 Factors associated with increased duration of milk OIT

#### Casey G. Cohen^1^, Bruce Mazer^1, 2^, Danbing Ke^2^, Wei W. Zhao^1^, Christine McCusker^2^, Julia Upton^4^, Eyal Grunebaum^4^, Edmond S. Chan^3^, Liane Beaudette^2^, Duncan Lejtenyi^2^, Ann E. Clarke^5^, Moshe Ben-Shoshan^2^

##### ^1^Research Institute of the McGill University Health Centre, McGill University, Montreal, QC, Canada; ^2^Division of Pediatric Allergy and Clinical Immunology, Department of Pediatrics, Montreal Children’s Hospital, Montreal, QC, Canada; ^3^Division of Allergy and Immunology, Department of Pediatrics, BC Children’s Hospital, University of British Columbia, Vancouver, BC, Canada; ^4^Division of Immunology and Allergy, Department of Pediatrics, The Hospital for Sick Children, University of Toronto, Toronto, ON, Canada; ^5^Division of Rheumatology, Department of Medicine, University of Calgary, Calgary, AB, Canada

**Correspondence:** Casey G. Cohen

*Allergy Asthma Clin Immunol* 2021, **17(Suppl 1):**12

**Background:** Food desensitization via Oral Immunotherapy (OIT) is gaining higher acceptance in clinical practice. Due to adverse reactions during OIT, the duration of the escalation phase until maintenance dose is achieved may be longer, and in a minority of cases, OIT is stopped. However, it is unclear what clinical factors are associated with longer duration of escalation phase. It is crucial to sort out these factors in order to allocate adequate resources for OIT. We aim to assess the clinical factors associated with longer milk OIT duration.

**Methods:** Data was collected from patients undergoing milk OIT at the Montreal Children’s Hospital, BC Children’s Hospital, and Hospital for Sick Children. We aimed to assess the duration of OIT with respect to reaching a maintenance dose of 200 mL of milk. We compared uni- and multivariable linear regressions to evaluate sociodemographic factors (age, sex), clinical characteristics (presence of co-morbidities such as asthma) and study entry variables (cumulative milk dose causing reaction, skin test wheal size, severity of entry challenge) associated with longer OIT duration.

**Results:** Among 51 children who reached 200 mL of milk, the median age was 12 years [Interquartile Range (IQR 9, 15)] and 54.9% were males. 86.3% of children had controlled asthma. The median cumulative dose of milk causing a reaction upon entry challenge was 14.4 mL (IQR 4.4, 44.4) and 21.6% received more than 1 dose of epinephrine. The median duration of escalation phase to reach 200 mL was 31 weeks (IQR 21.5, 39.8). Multiple doses of epinephrine (2 or more) during entry challenge was associated with longer OIT duration by almost 15 weeks [14.74, 95% CI: (2.75, 26.74)].

**Conclusions:** The data suggests the severity of adverse reactions at entry challenge is associated with longer escalation phase duration. Performing a challenge prior to OIT can assist in predicting the duration of OIT.

### #13 Skin prick test in milk allergic patients undergoing oral immunotherapy: randomized controlled crossover trial

#### Esraa Bukhari^1^, Sofianne Gabrielli^1^, Chris McCusker^1^, Duncan Lejtenyi^1^, Danbing Ke^2^, Bahar Torabi^2^, Moshe Ben-Shoshan^1^, Liane Beaudette^1^, Edmond S. Chan^3^, Julia Upton^4^, Eyal Grunebaum^4^, Ann Elaine Clarke^5^, Alexandra Langlois^6^

##### ^1^Division of Allergy and Clinical Immunology, Department of Pediatrics, Montreal Children’s Hospital, McGill University Health Centre, Montreal, QC, Canada; ^2^. he Research Institute of the McGill University Health Centre, Division of Pediatric Allergy and Clinical Immunology, Department of Pediatrics, Montreal Children’s Hospital, Montreal, QC, Canada; ^3^Division of Allergy and Immunology, Department of Pediatrics,BC Children’s Hospital, University of British Columbia, Vancouver, BC, Canada; ^4^Division of Immunology and Allergy, Department of Pediatrics, the Hospital for Sick Children, University of Toronto, Toronto, ON, Canada; ^5^Division of Rheumatology, Department of Medicine, University of Calgary, Calgary, AB, Canada; ^6^Division of Allergy and Clinical Immunology, Department of Pediatrics, Centre de recherche du centre hospitalier universitaire de Sherbrooke, University of Sherbrooke, Sherbrooke, QC, Canada

**Correspondence:** Esraa Bukhari

*Allergy Asthma Clin Immunol* 2021, **17(Suppl 1):**13

**Background:** Skin prick test (SPT) is the most commonly used confirmatory test for an IgE-mediated milk allergy. However, food SPTs are not standardized and it is not clear if commercial extracts are more appropriate than diluted/undiluted food in assessing food allergy resolution. We aimed to assess the accuracy of SPTs with extract, diluted (1:10), and undiluted milk to detect desensitization in children with milk allergy undergoing oral immunotherapy (OIT).

**Methods:** Children with milk allergy undergoing OIT and controls were recruited from Montreal Children’s Hospital (MCH), British Columbia Children’s Hospital (BCCH), and The Hospital for Sick Children (SickKids). Participants in the active arm received a weekly increase in milk until 200 ml of pure milk was tolerated. SPT using milk extract (Omega), diluted 2% milk (1:10), and undiluted milk was done at the study entry and when 200 ml of pure milk was reached. Participants in the control arm had SPT at study entry and 12 months later before they entered the active arm.

**Results:** Among 51 children who reached 200 ml, the median age was 12 years [Interquartile Range (IQR 9.25, 15.0)] and 52.9% were males. The mean decrease in wheal size at 200 ml from the baseline was 3.68 mm (95%CI 2.39–4.98), 5.01 mm (95% CI 3.57–6.44), and 4.97 mm (95% CI 3.12–6.82) for milk extract, diluted and undiluted milk respectively.

Among 32 controls, the median age was 10 years (IQR7.0, 14.25) and 62.5% were males. There was no significant change in wheal diameter over a one-year period regardless of the skin test method.

**Conclusions:** Response to extract behaved similarly to whole food (Diluted and undiluted) and thus can be used to follow sensitization in the context of a desensitization program.

### #14 Hidden allergens: an unusual case presentation of soy anaphylaxis in a 17-year-old male

#### Daniel H. Li^1^, Hannah T. Roberts^2^

##### ^1^Department of Internal Medicine, University of Toronto, Toronto, ON, Canada; ^2^Division of Allergy and Immunology, Department of Medicine, Western University, London, ON, Canada

**Correspondence:** Daniel H. Li

*Allergy Asthma Clin Immunol* 2021, **17(Suppl 1):**14

**Background:** Soy allergy is a common IgE-mediated food allergy in young children, but rarely presents for the first time in adolescence. Recent studies have shown that approximately 50% of children with soy allergy outgrow their allergy by age 7 [1]. We present a unique case of new onset soy allergy in a 17-year-old.

**Case Presentation:** A 17-year-old male was referred for anaphylaxis to an unknown trigger. He consumed a smoothie and immediately developed diffuse urticaria, facial angioedema, wheeze, and chest tightness. He was taken to the emergency department via ambulance and treated with epinephrine. On review, the store-bought “fruit smoothie” contained apple, pineapple, orange, banana, soy and whey protein. There were no cofactors. After the episode, he consumed all fruits and milk without reaction, but not soy. Prior to anaphylaxis, he was not actively avoiding soy protein and he consumed soy in infrequent quantities. Interestingly, he also has a chickpea allergy, but the index reaction was at age 6. He did not avoid other legumes for cross-reactivity concerns. Skin prick testing was performed with appropriate controls. Whey protein was negative. Soy protein was strongly positive at 10 × 8 mm. Serum specific immunoglobulin E (IgE) was elevated to soybean at 12.92 kilounits/litre and whey was negative.

**Conclusions:** It is highly unusual for a patient at this age to present with a new IgE-mediated soy allergy. This case is important as it highlights the potential for hidden allergens in “organic health foods”. Our patient had a fruit smoothie without realizing it was a soy protein containing beverage and developed unexpected anaphylaxis. Although this patient was unaware of his new soy allergy, the case exemplifies why it is crucial to educate patients on the importance of reading labels. This case will contribute to food allergy literature, especially soy and legume allergy.

**Statement of Consent:** Written informed consent for this case report was obtained from the patient.

### #15 The impact of COVID-19 on food-allergy-specific anxiety: a cross-sectional survey of parents of children with food allergies

#### Clara Westwell-Roper^1,2^, Sharon To^3^, Lianne Soller^3^, S. Evelyn Stewart^2,4,5^, Edmond S. Chan^3,5^

##### ^1^British Columbia Children’s Hospital Research Institute, Vancouver, BC, Canada; ^2^Division of Clinical and Behavioural Neurosciences, Department of Psychiatry, University of British Columbia, Vancouver, BC, Canada; ^3^Division of Allergy and Immunology, Department of Pediatrics, Faculty of Medicine, University of British Columbia, Vancouver, BC, Canada; ^4^British Columbia Mental Health and Substance Use Research Institute, Vancouver, BC, Canada; ^5^SES and ESC are joint senior authors. British Columbia Children’s Hospital Research Institute, Vancouver, BC, Canada

**Correspondence:** Clara Westwell-Roper

*Allergy Asthma Clin Immunol* 2021, **17(Suppl 1):**15

**Background:** Parenting a child with food allergy (FA) is associated with reduced health-related quality of life (HRQOL), including FA-specific anxiety (FAA). We characterized effects of the COVID-19 pandemic on FAA among parents of children with FA in Canada.

**Methods:** A survey link was distributed by Food Allergy Canada and the Metro Vancouver Anaphylaxis Group in May–June 2020. The survey included medical/demographic information, anxiety and HRQOL measures, a 31-item FAA questionnaire undergoing validation (querying current/pre-COVID-19 symptoms), and COVID-19 impact rating scales. Groups were compared by two-tailed *t* test, one-way ANOVA, or Chi squared test. Reflexive thematic analysis was applied to open-ended responses.

**Results:** 293 consenting participants completed the FAA questionnaire. 92% were mothers, reporting a greater share of management responsibility than fathers (78.5% ± 17.6% vs. 57.6% ± 23.0%, *p *< 0.0001). 66.7% reported increased overall anxiety due to COVID-19. Only 28.1% reported increased FAA (*p *< 0.0001), which was unchanged (29.5%) or decreased (42.3%) among remaining respondents. Among those with increased overall anxiety attributed to COVID-19, FAA was decreased or unchanged in 35.4% and 24.0%, respectively. Global FAA correlated weakly with general anxiety measures (GAD-7 *ρ *= 0.273; STAI-S *ρ *= 0.371) and moderately with FA-specific HRQOL (FAQL‑PB *ρ *= 0.688; all *p *< 0.0001). More respondents with past FA-related emergency visits reported COVID-19-related FAA than those without (33.0% vs. 16.0%, *p *= 0.0041). COVID-19 was associated with a decrease in all FAA dimensions (emotions, cognitions, physical symptoms, behaviours, and coping) and their functional impacts. Qualitative themes included both positive and negative impacts: decreased worry about out-of-home allergen exposures, lack of “safe” food availability, concern about health care system FA management capacity; and risks of infection associated with emergency care.

**Conclusions:** Despite increased overall anxiety, most parents reported unchanged or decreased FAA associated with COVID-19-related restrictions. Further studies should evaluate methods for identifying families requiring mental health support for FA management, particularly as restrictive guidelines are relaxed and perceived allergen exposure risk increases.

### #16 Erythromycin causing peanut-induced allergic reaction during oral immunotherapy

#### Joel Liem^1,2^, Eunice Tunggal^1^

##### ^1^Windsor Asthma Allergy Education Centre, Oakville, ON, Canada; ^2^Schulich School of Medicine and Dentistry, Western University, London, ON, Canada

**Correspondence:** Eunice Tunggal

*Allergy Asthma Clin Immunol* 2021, **17(Suppl 1):**16

**Background:** Erythromycin is an antibiotic used as an alternative medication for patients with penicillin allergies. Recent studies determined that erythromycin mimics motilin, stimulating motilin receptors in the gut. Duodenal smooth muscles contract, ultimately accelerating food absorption.

**Case Presentation:** We report a case of allergic reaction during peanut oral immunotherapy (OIT) associated with erythromycin use.

A 17-year-old female undergoing peanut OIT was prescribed erythromycin 500-mg po QID for persistent sore throat. She has history of penicillin allergy. She was taking 75-mg of peanut protein each day for 7 days prior, without reaction. She took her first erythromycin dose the night before, feeling nauseous by morning. She took her second and third erythromycin doses by 11:00AM, then took 75-mg OIT dose at 4:15PM. 45 min post-OIT-dose, she felt intensified nausea and stomach pain. 1.5 h post-OIT-dose, she developed facial hives which gradually spread systemically, and severe nausea/stomach pain. She had not exercised, taken the protein on an empty stomach, or had a fever before taking dosage. She was taken to hospital and treated with Benadryl and prednisone. Erythromycin was stopped. She was challenged to 75-mg protein the next day. No reaction.

**Conclusions:** Erythromycin use during OIT treatment increases reaction risk. This case may aid allergists conducting OIT programs to be wary of risks to patients using erythromycin and fellow reported motilin agonists; macrolide antibiotics- including clarithromycin, azithromycin, roxithromycin, and oleandomycin- during treatment. Of clinical importance is recognizing that erythromycin induces GI hypermotility and may cause reactions to OIT doses due to accelerated protein dose absorption.

**Statement of Consent:** Written informed consent for this case report was obtained from the patient.

### #17 Low use of epinephrine in management of anaphylaxis occurring in restaurants

#### Hoang Pham^1^, Jordan Trevisonno^2^, Sofianne Gabrielli^3^, Ann Clarke^4^, Judy Morris^5^, Adam Bretholz^6^, Ran D. Goldman^7^, Edmond S. Chan^8^, Andrew O’Keefe^9^, Robert Porter^10^, Julia Upton^11^, Elana Hochstadter^12^, Jennifer Gerdts^13^, Derek Chu^16^, Jennifer L. Protudier^17,18,19^, Elissa M. Abrams^18,19^, Elinor Simons^19^, Moshe Ben-Shoshan^3^

##### ^1^Division of Clinical Immunology and Allergy, Department of Medicine, McGill University Health Centre, Montreal, QC, Canada; ^2^Department of Medicine, Montreal General Hospital, McGill University Health Centre, Montreal, QC, Canada; ^3^Division of Allergy and Clinical Immunology, Department of Pediatrics, Montreal Children’s Hospital, McGill University Health Centre, Montreal, QC, Canada; ^4^Division of Rheumatology, Department of Medicine, Cummings School of Medicine, University of Calgary, Calgary, AB, Canada; ^5^Department of Emergency Medicine, Sacré-Coeur Hôpital, Montreal, QC, Canada; ^6^Department of Emergency Medicine, Montreal Children’s Hospital, McGill University Health Centre, Montreal, QC, Canada; ^7^Division of Clinical Pharmacology and Emergency Medicine, Department of Pediatrics, BC Children’s Hospital, University of British Columbia, Vancouver, BC, Canada; ^8^Division of Allergy and Immunology, Department of Pediatrics, BC Children’s Hospital, University of British Columbia, Vancouver, BC, Canada; ^9^Division of Pediatrics, Faculty of Medicine, Memorial University of Newfoundland, St. John’s, NL, Canada; ^10^Division of Emergency Medicine, Faculty of Medicine, Memorial University of Newfoundland, St. John’s, NL, Canada; ^11^Division of Immunology and Allergy, Department of Pediatrics, Hospital for Sick Children, University of Toronto, Toronto, ON, Canada; ^12^Division of Pediatric Emergency Medicine, Department of Pediatrics, Hospital for Sick Children, University of Toronto, Toronto, ON, Canada; ^13^Food Allergy Canada, National Patient Organization, Toronto, ON, Canada; ^14^Department of Medicine and Health Research Methods, Evidence, & Impact, McMaster University, Hamilton, ON, Canada; ^15^The Research Institute of St. Joe’s Hamilton, Hamilton, ON, Canada; ^16^Department of Medicine and Health Research Methods, Evidence, & Impact, McMaster University and The Research Institute of St. Joe’s Hamilton, Hamilton, ON, Canada; ^17^ Department of Pediatrics and Child Health, University of Manitoba, Winnipeg, MB, Canada; ^18^ Children’s Hospital Research Institute of Manitoba, Winnipeg, MB, Canada; ^19^George and Fay Yee Centre for Healthcare Innovation, Winnipeg, MB, Canada

**Correspondence:** Hoang Pham

*Allergy Asthma Clin Immunol* 2021, **17(Suppl 1):**17

**Background:** The prevalence of food allergy has remained stable [1], but the rate of anaphylaxis has increased over the last decade [2]. Reactions may occur in different settings, including restaurants. There is lack of prospective data assessing the management of anaphylaxis in restaurants.

**Methods:** We assessed cases of anaphylaxis occurring in restaurants through the Cross-Canada Anaphylaxis Registry, a cohort study established in 2011 that enrolls children and adult anaphylaxis cases of presenting to emergency departments in 5 Canadian provinces. Participants were recruited prospectively and retrospectively using ICD10 codes. Data were collected on baseline sociodemographic and clinical characteristics as well as location, clinical manifestations, and management of reactions using a standardized questionnaire. Cases were classified as mild, moderate, or severe according to the position paper of the European Academy of Allergology and Clinical Immunology on management of anaphylaxis in childhood [3]. Multivariate logistical regression was used to identify factors associated with epinephrine use in restaurants.

**Results:** Among 187 cases of anaphylaxis occurring in restaurants, 73.3% involved children. Reactions were mainly triggered by food (94.7%) while the remainder were triggered by drug (0.5%) or unknown cause (4.8%). Of the reactions with known triggers, the most common triggers were peanut (18.2%), shellfish (9.6%), and tree nut (8%). Pre-hospital epinephrine use was documented in 39% of the cohort and in 48% of those with known food allergies. Epinephrine use in restaurants was more likely in cases with known food allergy (OR 1.12; 95% CI 1.12–1.50) and for moderate/severe reactions (OR 1.24; 95% CI 1.03–1.50).

**Conclusions:** Epinephrine was not used in the majority of anaphylaxis occurring in restaurants, even among those with known food allergy. Educational programs promoting the use of epinephrine as well as policies encouraging restaurants to stock and train restaurant staff to use epinephrine auto-injectors are required.

**References**

1. Clarke AE, Elliott SJ, Pierre YS, Soller L, La Vieille S, Ben-Shoshan M. Temporal trends in prevalence of food allergy in Canada. J Allergy Clin Immunol Pract. 2020;8(4):1428–30.e5.
2. Hochstadter E, Clarke A, De Schryver S, LaVieille S, Alizadehfar R, Joseph L, Eisman H, Ben-Shoshan M.J. Increasing Visits for Anaphylaxis and the Benefits of Early Epinephrine Administration: A 4-year Study at a Pediatric Emergency Department in Montreal, Canada. J Allergy Clin Immunol. 2016;137(6):1888–90.e4.
3. Muraro A, Roberts G, Clark A, et al. The management of anaphylaxis in childhood: position paper of the European academy of allergology and clinical immunology. Allergy. 2007;62(8):857–71.

### #18 Retrospective case series of patients with Peri-Operative Anaphylaxis (POA) at a major Canadian teaching hospital

#### Juan C. Ruiz^1^, Julena F. Foglia^2^, Raymond Mak^1^, Andrew Meikle^2^, Tim T. Lau^3^, Amin Kanani^1^

##### ^1^Division of Allergy and Immunology, Department of Medicine, University of British Columbia, Vancouver, BC, Canada; ^2^Department of Anesthesia, University of British Columbia, Vancouver, BC, Canada; ^3^Pharmaceutical Sciences, Vancouver General Hospital, and Faculties of Pharmaceutical Sciences and Medicine, University of British Columbia, Vancouver, BC, Canada

**Correspondence:** Juan C. Ruiz

*Allergy Asthma Clin Immunol* 2021, **17(Suppl 1):**18

**Background:** Perioperative anaphylaxis (POA) is a life-threatening hypersensitivity reaction during surgery or an invasive procedure. Diagnosing POA is challenging as patients may present atypically. Currently, data on the prevalence of POA and its causative agents are based on the NAP6, which is the largest study derived in the UK. To our knowledge, there is limited published Canadian data on POA.

**Methods:** A retrospective chart review was performed for patients seen at the Vancouver General Hospital Perioperative Anaphylaxis Clinic between November 2018 to November 2019. Patients were included based on the 2019 Consensus Clinical Scoring for Suspected Perioperative Immediate Hypersensitivity Reactions. The culprit for the reaction was identified through positive skin testing using non-irritant concentrations.

**Results:** 10 out of 29 patients assessed in our clinic met inclusion criteria. Average score was 20, indicating a high likelihood of reaction. Commonly reported clinical presentations were hypotension defined as systolic blood pressure of < 90 mmHg (N = 10), followed by bronchospasm (N = 4), and angioedema, and urticaria (N = 4). Other more severe presentations included cardiac arrest (N = 2). Acute tryptase was sent in 7 cases and was elevated in 6 cases. Identified triggers were cefazolin (N = 4), chlorhexidine (N = 4), patent blue dye (N = 1), and bacitracin (N = 1). For treatment, all patients received epinephrine.

**Conclusions:** Hypotension appears to be the most common presentation associated with POA. Frequent triggers included cefazolin and chlorhexidine. Our findings are consistent with US data supporting antibiotics as the main cause of POA. Furthermore, similar to the UK, chlorhexidine POA is becoming more prevalent. In contrast to European data, no cases of neuromuscular blocking agent or latex anaphylaxis were observed. We highlight initial epidemiological data, which suggests differences in the specific causative agents of POA in British Columbia. Further studies are necessary to characterize Canadian POA epidemiology.

### #19 Associations between age and the safety and efficacy of peanut oral immunotherapy

#### Rongbo Zhu^1,2^, Jennifer L. Protudjer^3,4,5^, Kara Robertson^1,2^, Andrea Macikunas^1^, Rebecca Kim^6^, Samira Jeimy^1,2^, Harold Kim^1,2,7^

##### ^1^Schulich School of Medicine & Dentistry, Western University, London, ON, Canada; ^2^Division of Clinical Immunology and Allergy, Department of Medicine, Western University, London, ON, Canada; ^3^Department of Pediatrics and Child Health, University of Manitoba; George and Fay Yee Centre for Healthcare Innovation; Children’s Hospital Research Institute of Manitoba, Winnipeg, MB, Canada; ^4^Department of Food and Human Nutritional Sciences, Winnipeg, MB, Canada; ^5^Institute for Environmental Medicine, Karolinska Institutet, Stockholm, Sweden; ^6^Queen’s University Faculty of Arts and Science, Kingston, ON, Canada; ^7^Department of Medicine, McMaster University, Hamilton, ON, Canada

**Correspondence:** Rongbo Zhu

*Allergy Asthma Clin Immunol* 2021, **17(Suppl 1):**19

**Background:** Peanut oral immunotherapy (POIT) is an effective treatment in peanut allergic children which improves quality of life. POIT is efficacious and safe in preschool-aged and older children, with some speculation that age-related differences in efficacy and safety may exist. However, these differences have not been studied via a standardized protocol. The aim of our study is to examine associations between age at start of POIT, and the efficacy and safety of POIT, using a standardized protocol.

**Methods:** This is a single-center retrospective study of pediatric patients (< 18 years old), conducted between June 2016 and November 2019. Patients underwent monthly clinic visits over 14 months, and peanut dosing was escalated to 250 mg of peanut protein using formulary capsules containing peanut flour. Completion of POIT was defined by tolerance of at least 250 mg of peanut protein in capsules, peanut butter, or peanut M&Ms. Safety was defined as epinephrine use during POIT. Data were analyzed using n/N, %, mean ± standard deviation, and binary logistic regression analysis (OR, 95% CI).

**Results:** Overall, 158 patients (61.4% male; mean age 8.40 ± 4.25 years) were included. Mean baseline skin prick test (SPT) for peanut was 8.49 ± 3.94 mm. Mean baseline peanut IgE was 50.79 ± 41.06 kU/L. In addition to peanut allergy, 41.2% (65/158) had at least one other food allergy and 82.3% (130/158) had comorbid allergic disease. In total, 70.9% (112/158) patients completed per protocol, and 5.1% (8/158) required epinephrine during POIT. Older age at baseline was inversely associated with the odds of completion (OR 0.70; 95% CI 0.54–0.92), whereas no association was found between age and epinephrine during OIT (OR 0.49; 95% CI − 0.97–1.95).

**Conclusions:** Younger children are significantly more likely to successfully complete POIT using a standardized protocol, whereas the safety of POIT is not significantly associated with age.

### #20 Adult and pediatric food allergy to non-priority plant proteins in the clinical context: a scoping review

#### Hailey V. Hildebrand^1^, Elinor Simons^2,3^, Jennifer Gerdts^4^, Beatrice Povolo^4^, Ana T. Arias^5^, Janet Rothney^6^, Jennifer L. Protudjer^2,3,7^

##### ^1^Department of Medicine, University of Manitoba, Winnipeg, MB, Canada; ^2^Department of Pediatrics and Child Health, University of Manitoba, Winnipeg, MB, Canada; ^3^The Children’s Hospital Research Institute of Manitoba, Winnipeg, MB, Canada; ^4^Food Allergy Canada, Toronto, ON, Canada; ^5^Centro Universitario de Ciencias de la Salud, Universidad de Guadalajara, Guadalajara, JAL, Mexico; ^6^Neil John Maclean Health Sciences Library, University of Manitoba, Winnipeg, MB, Canada; ^7^Institute of Environmental Medicine, Karolinska Institutet, Stockholm, Sweden

**Correspondence:** Hailey V. Hildebrand

*Allergy Asthma Clin Immunol* 2021, **17(Suppl 1):**20

**Background:** Plant-based diets are becoming increasingly popular, resulting in an increased demand for plant-based protein substitutes, including chickpea, pea, lentil, and lupine. Recently, these legumes have been identified as the cause of allergic reactions and in some cases, anaphylaxis. However, there is limited knowledge about these emerging allergens, and the potential impact they may have on the consumer and on the food industry. The aim of this study is to summarize the available evidence on non-priority plant protein food allergy in the clinical context.

**Methods:** Our scoping review will follow the PRISMA-ScR checklist and the Arksey and O’Malley framework, and will search MEDLINE, EMBASE, CINAHL, Scopus, and CENTRAL. The research question we intend to answer is “What is known from the existing literature about non-priority plant protein food allergy, including but not limited to, chickpea, pea, lentil, and lupine, but excluding peanut and soy, in the clinical context?” The study population includes all ages. Inclusion criteria will include peer-reviewed primary literature in English, French, Spanish, and Swedish languages, regardless of the country of study and date published. The gray literature will be searched, including the bibliographies of accepted full-articles, abstracts from relevant conferences, and associated government policies. We will exclude non-clinical and preclinical studies, as we want to determine how non-priority plant protein food allergy affects patients. All screening and data charting will be performed by two independent reviewers, with disagreements solved by decision of a third reviewer.

**Results:** Our comprehensive literature search retrieved 5644 unique records. We anticipate this scoping review will provide relevant information on novel non-priority plant protein sources as they are related to food allergy.

**Conclusions:** In the future, this review will be used in combination with patient engagement to inform roundtable discussions with food manufacturers, health policy makers, and our national patient advocacy organization, Food Allergy Canada.

### #21 Exploring epinephrine use by food-allergic families during COVID-19 pandemic in British Columbia

#### Lianne Soller, Scott B. Cameron, Edmond S. Chan

##### UBC, Vancouver, BC, Canada

**Correspondence:** Lianne Soller

*Allergy Asthma Clin Immunol* 2021, **17(Suppl 1):**21

**Background:** There is no research on how COVID-19 has affected rate of epinephrine autoinjector (EAI) use among food-allergic patients. We hypothesized that quarantine during COVID-19 would lead to lower rates of accidental exposure and subsequent use of EAI due to fewer opportunities for leaving the home and fewer instances of a non-primary caregiver preparing food. This study sought to determine whether COVID-19 affected the rate of EAI administration in food-allergic families.

**Methods:** An online survey was sent to BC patients who had allergist confirmed food allergy, asking whether EAI was administered in the past year pre-COVID-19 or during COVID-19. The COVID-19 period included 4 months (33.3%) of the year (Mar/2020–Jun/2020) and the pre-COVID-19 period included 8 months (66.7%) of the year (Jul/2019–Feb/2020). A two-sample t-test was used to compare the proportion of patients administering EAI during COVID-19 with the proportion of the year that COVID-19 quarantine was in place.

**Results:** Between May–Jun/2020, 66 of 309 (Response rate 21.4%) families completed the COVID-19 questionnaire. Of these, 8/66 (12.1%) administered EAI in the past year, with 1/8 (12.5%) during COVID-19. Had distribution been uniform and unchanged, we would expect instead 33.3% of episodes to occur during COVID-19. However, the t-test did not show a significant difference (p = 0.15) between these two proportions.

**Conclusions:** This is the first study to examine accidental exposure requiring EAI during COVID-19. Although the proportion administering EAI during COVID-19 was less than expected, this was non-significant, possibly due to small sample size. Seasonal variation in anaphylaxis could also explain the rate of EAI during COVID-19. More studies with a larger sample size are needed to draw definitive conclusions about whether COVID-19 had an impact on the use of EAI by food-allergic families.

### #22 Salmon allergy: wild vs farmed: is there a difference?

#### Wardha Wardha^1^, Jason A. Ohayon^1,2^

##### ^1^Hamilton Allergy, Hamilton, ON, Canada; ^2^McMaster University, Hamilton, ON, Canada

**Correspondence:** Wardha Wardha

*Allergy Asthma Clin Immunol* 2021, **17(Suppl 1):**22

**Background:** Fish allergy, both finned and shellfish has increased in recent years with prevalence rates of self-reported allergy ranging from 0.2% to 2.29% in the general population. The major allergy-eliciting protein in salmon is parvalbumin, a calcium-binding protein from the albumin family with a low molecular weight that is found in the white muscle.

**Case Presentation:** A 4-year-old presented to a local community clinic with a prior history of generalized urticaria and agitation after consumption of salmon. Skin prick testing (SPT) revealed negative responses to both extract and cooked salmon. He subsequently passed an oral food challenge (OFC) in which he consumed 7.5 g of salmon and was declared salmon tolerant.

Four months later, he developed urticarial lesions and pruritus upon having a salmon dish at a restaurant. The reaction was successfully treated with diphenhydramine. Subsequent SPT to salmon revealed a negative response to salmon extract and a positive response to both raw (9 mm) and cooked (10 mm) wild Atlantic salmon. Patient went on to successfully pass a second OFC to farmed salmon and was recommended to avoid wild Atlantic salmon.

**Conclusions:** Based on current research, wild Atlantic salmon has a significantly larger muscle mass in comparison to farmed salmon. It is hypothesized that the patient developed an allergic reaction to wild Atlantic salmon because of increased exposure to parvalbumin in the higher concentrated protein form of salmon. However, further research is needed to elucidate the possibility of other allergenic proteins unique to the Atlantic salmon wild from farmed salmon. In addition, this report indicates the importance of testing with forms of the suspected allergen, beyond extract alone, to include both raw and cooked forms.

### #23 Not the high you wanted: Risk of cannabis induced anaphylaxis in patients with pollen food allergy syndrome

#### Sofiya Portuhay^1,2^, Jason Ohayon^2^

##### ^1^McMaster University, Hamilton, ON, Canada; ^2^Hamilton Allergy, Hamilton, ON, Canada

**Correspondence:** Sofiya Portuhay

*Allergy Asthma Clin Immunol* 2021, **17(Suppl 1):**23

**Background:** Plant food allergies are often related to inhalant allergens, such as grasses, trees and weeds. This relationship can be attributed to cross-reactive proteins. An example of this is birch tree and certain tree nuts, such as almond and hazelnut. Cross reactivity between cannabis and plant proteins has been reported. Based on available data, this link is due to non-specific lipid transfer proteins (LTP).

**Case Presentation:** A 19 year old male with significant pollen allergies had a history of tree nut tolerance, with occasional mild oral pruritus to raw nuts. The patient inhaled cannabis and consumed a nut bar, which contained almond, sesame and rice. Soon after, he developed systemic urticaria and wheezing, requiring emergency treatment for anaphylaxis. In follow-up, skin prick testing (SPT) identified strongly positive responses to grasses and ragweed. SPT was positive to almond and hazelnut, despite previous clinical tolerance, and negative to sesame. The patient underwent an in office almond oral challenge and was able to tolerate initial dosing of 15 grams. Anaphylaxis occurred at a cumulative dose of 30 grams, characterized by systemic urticaria, dysphagia, nausea and rhinorrhea. He was treated with epinephrine, prednisone, saline and allergy medications. He was advised to carry epinephrine and to avoid almonds.

**Conclusions:** The above patient experienced anaphylaxis to almond on two occasions; after cannabis inhalation and once consuming the final oral challenge dose. The initial anaphylaxis may have been facilitated by the preceding cannabis. Subsequent in office challenge at higher threshold dosing confirmed his almond allergy. It is hypothesized that cannabis inhalation may have reduced the threshold of anaphylaxis to almond, due to “allergic” LTP association. With the legalization of cannabis, the possibility of similar reactions occurring is increased. Further research on the effect of cannabis in relation to various food sensitization in the pollen food syndrome population is needed.

**Statement of Consent:** Written informed consent for this case report was obtained from the patient.

### #24 The burden of non-priority legume allergy amongst families of children and adults with multiple food allergies

#### Hailey V. Hildebrand^1^, Elissa M. Abrams^2,5^, Jennifer Gerdts^3^, Beatrice Povolo^3^, Harold Kim^4,6^, Jennifer L. Protudjer^5,2,7^

##### ^1^Department of Medicine, University of Manitoba, Winnipeg, MB, Canada; ^2^Department of Pediatrics and Child Health, University of Manitoba, Winnipeg, MB, Canada; ^3^Food Allergy Canada, Toronto, MB, Canada; ^4^Department of Clinical Immunology and Allergy, Western University, London, ON, Canada; ^5^The Children’s Hospital Research Institute of Manitoba, Winnipeg, MB, Canada; ^6^Department of Clinical Immunology and Allergy, McMaster University, Hamilton, ON, Canada; ^7^Institute of Environmental Medicine, Karolinska Institutet, Stockholm, Sweden

**Correspondence:** Hailey V. Hildebrand

*Allergy Asthma Clin Immunol* 2021, **17(Suppl 1):**24

**Background:** Food allergy is associated with decreased quality of life. Although Canadian labelling regulations require priority allergens to be listed if used as a component of an ingredient, there are no such requirements for non-priority allergens, including legumes. We define legume allergy as the non-priority allergens, chickpea, pea, lentil, and lupine, but excluding peanut and soy. Legume use in food products has increased as the shift towards plant-based diets gains popularity. We aimed to examine burden of legume allergy in Canadians with multiple food allergies.

**Methods:** This study uses data from MultidimeNsional bUrden of Allergies iN Canadian childrEn and adultS (NUANCES), an online survey of adults/families with multiple food allergies. Our study population was restricted to participants who reported sesame allergy as one of the foods to which they were allergic. Families reporting only a single food allergy were excluded. Data were described using n/N and percentage (%). This study was approved by The University of Manitoba Health Research Ethics Board (HS23019(H2019:317)).

**Results:** NUANCES included 192 participants (126 [65.6%] children), of whom all children and all but 2 adults (64/66; 97.0%) were allergic to 1 + priority allergens. A total of 38/66 (57.6%) adults and 41/126 (32.5%) children reported allergies to non-priority foods. Of these, 12/38 (31.6%) adults and 15/41 (36.6%) children were allergic to legumes; this constituted our study population. Nearly all with legume allergy also reported peanut allergy (15/15 [100%] children; 11/12 [91.7%] adults). Whereas legume allergy was rarely a source of social burden or worry in restaurants, approximately half of adults (5/12; 41.7%) and families (6/11; 54.6%) reported that legumes were the allergy for which they wanted ingredient information.

**Conclusions:** Adults/families of children with multiple food allergies, including legumes, report a need for greater ingredient disclosure for non-priority allergens, but describe limited social burden associated with legume allergy.

### #25 Characterization of boney fish allergy and its relationship with atopic dermatitis in Manitoba children

#### Bitian Meng^1^, Paria Kian^1^, Jennifer Protudjer^1,2,3^, Elinor Simons^3,4^

##### ^1^Rady Faculty of Health Sciences, Winnipeg, MB, Canada; ^2^Department of Pediatrics and Child Health, Winnipeg, MB, Canada; ^3^Children’s Hospital Research Institute of Manitoba, Winnipeg, MB, Canada; ^4^Section of Allergy and Clinical Immunology, Winnipeg, MB, Canada

**Correspondence:** Bitian Meng

*Allergy Asthma Clin Immunol* 2021, **17(Suppl 1):**25

**Background:** Boney fish allergy affects 1% of Canadian children, is common in Manitoba and is rarely outgrown. Atopic dermatitis (AD) is the most common inflammatory skin condition in children and has been increasingly documented to be associated with food allergy development. We examined boney fish allergy and its relationship with AD among Manitoba children.

**Methods:** This retrospective study longitudinally examined a cohort of children with boney fish allergy followed in Pediatric Allergy Clinic. We collected data from patient charts regarding age of boney fish allergy diagnosis, sensitization measured by skin prick testing or specific IgE level, allergy resolution, and presence and severity of AD. We also recorded the child’s gender, history of allergy to foods other than boney fish and family history of atopic conditions. We used multivariable logistic regression to examine the association between resolution of boney fish allergy and moderate-to-severe AD, after adjusting for potential confounders.

**Results:** Of the 297 children with boney fish allergy (median age 4.3 years, range 4.7 months-18 years), 39.1% were female, 69.7% were sensitized to multiple types of boney fish, 67.3% had AD, 28.7% had moderate-to-severe AD, 50.8% had asthma and 64.3% were allergic to foods other than boney fish. Overall, 33 children had improvement of their AD until it was no more than mild. Among the 6 children who outgrew their fish allergy, 4 (2.5%) had absent-to-mild AD and 2 (1.9%) had moderate-to-severe AD. Children who outgrew their boney fish allergy were more likely to be sensitized to only one type of boney fish (p = 0.04). After adjusting for boney fish monosensitization, children with moderate-to-severe AD were no less likely to outgrow their boney fish allergy (OR 1.17, 95% CI 0.21–6.63).

**Conclusions:** Children who outgrew their boney fish allergy were more likely to be monosensitized but no more likely to have moderate-to-severe AD.

### #26 Pediatric anaphylaxis visits to the emergency department during COVID-19

#### Daniel Sehayek^1^, Morgan S. Gold^2^, Sofianne Gabrielli^3^, Ann E. Clarke^4^, Christine McCusker^3^, Xun Zhang^5^, Judy Morris^6^, Rodrick Lim^7^, Edmond S. Chan^8^, Ran D. Goldman^9^, Andrew O-Keefe^10^, Jennifer Gerdts^11^, Derek K. Chu^12^, Julia Upton^13^, Adam Bretholz^14^, Moshe Ben-Shoshan^3^

##### ^1^Faculté de Médecine, Université Laval, Quebec City, QC, Canada; ^2^Faculty of Medicine, McGill University, Montreal, QC, Canada; ^3^Division of Pediatric Allergy and Clinical Immunology, Department of Pediatrics, McGill University Health Centre, Montreal, QC, Canada; ^4^Division of Rheumatology, Department of Medicine, Cumming School of Medicine, University of Calgary, Calgary, AB, Canada; ^5^Centre for Outcomes Research and Evaluation, Research Institute of McGill University Health Centre, Montreal, QC, Canada; ^6^Department of Emergency Medicine, Sacré-Coeur Hôpital, Montreal, QC, Canada; ^7^Division of Pediatric Emergency Medicine, Department of Pediatrics, Children’s Hospital at London Health Science Centre, London, ON, Canada; ^8^Division of Allergy and Immunology, Department of Pediatrics, BC Children’s Hospital, University of British Columbia, Vancouver, BC, Canada; ^9^Division of Clinical Pharmacology and Emergency Medicine, Department of Pediatrics, BC Children’s Hospital, University of British Columbia, Vancouver, BC, Canada; ^10^Division of Allergy and Immunology, Department of Pediatrics, Faculty of Medicine, Memorial University, St. John’s, NL, Canada; ^11^Food Allergy Canada, Toronto, ON, Canada; ^12^Division of Clinical Immunology & Allergy, Department of Medicine, and Department of Health Research Methods, Evidence & Impact, McMaster University, Hamilton, ON, Canada; ^13^Division of Immunology and Allergy, Department of Pediatrics, The Hospital for Sick Children, Department of Paediatrics, University of Toronto, Toronto, ON, Canada; ^14^Department of Emergency Medicine, Montreal Children’s Hospital, Montreal, QC, Canada

**Correspondence:** Daniel Sehayek

*Allergy Asthma Clin Immunol* 2021, **17(Suppl 1):**26

**Background:** COVID-19 has caused unprecedented fear of visiting public places, especially hospitals. As such, we assessed whether the rate of visits to a pediatric emergency department (ED) for anaphylaxis in 2020 had decreased compared to 2019.

**Methods:** Children presenting to the Montreal Children’s Hospital ED from February 2019 to May 2020 with anaphylaxis were recruited for the Cross-Canada Anaphylaxis REgistry (C-CARE). A standardized form documenting symptoms, triggers and management was collected. Total number of anaphylaxis visits were compared by an unpaired Wilcoxon test.

**Results:** Between February and May 2019, there were 26,570 ED visits, 98 (0.37%) of which were anaphylaxis. Of these cases [median age 9.1, Interquartile Range (IQR) = 13.78, 3.25], 91 (92.86%) were food-induced and 16 (16.33%) were severe. 49 (50%) and 50 (51.02%) patients received out-of-hospital and in-hospital epinephrine, respectively. Between February and May 2020, there were 13,960 ED visits, 54 (0.39%) of which were anaphylaxis. Of these cases (median age 3.5, IQR = 8.83, 1.58), 49 (90.74%) were food-induced and 2 (3.70%) were severe. 34 (62.97%) and 20 (37.04%) patients received out-of-hospital and in-hospital epinephrine, respectively.

Between these months in 2019 and 2020, there was no significant difference in proportion of overall and monthly anaphylaxis cases among all ED visits. Excluding February from the analysis did not change the significance. Furthermore, there was no significant difference in food-induced anaphylaxis. However, there was a significant difference in severity, with 12.62% (95%CI: 2.30%, 22.94%) less in 2020.

**Conclusions:** The decreased rate of severe reactions could show hesitancy to bring children with severe reactions to the ED. However, it is also possible that restricted social interactions and increased parental supervision, which increased supervised food introduction, reduced severe accidental reactions. The lower median age may indicate that only parents of younger children believe the risk of anaphylaxis outweighs that of COVID-19.

### #27 Is atopic dermatitis associated with delayed resolution of egg allergy in infants?

#### Amarjot Padda^1^, Mihaela Paina^2^, Elinor Simons^2^

##### ^1^Department of Pediatrics and Child Health, University of Manitoba, Winnipeg, MB, Canada; ^2^Section of Allergy and Immunology, Department of Pediatrics and Child Health, University of Manitoba, Winnipeg, MB, Canada

**Correspondence:** Amarjot Padda

*Allergy Asthma Clin Immunol* 2021, **17(Suppl 1):**27

**Background:** Atopic dermatitis affects 20% of children and 50% is diagnosed by age 1 year; 1% of children have egg allergy and 50% of egg allergy is outgrown. According to the dual allergen hypothesis, oral antigen exposure promotes tolerance while epicutaneous exposure promotes sensitization. Early, persistent, and severe atopic dermatitis has been associated with developing egg allergy. We hypothesize that infants with egg allergy are more likely to outgrow their allergy if they do not have atopic dermatitis.

**Methods:** We reviewed charts of all infants with egg allergy referred to Pediatric Allergy Clinic in the first year of life and evaluated from 2013–2017. We collected data regarding egg allergy diagnosis, development of egg and baked egg tolerance, and the presence, severity and control of atopic dermatitis. We used multivariable logistic regression to evaluate the association between egg allergy resolution and presence of atopic dermatitis.

**Results:** Of the 83 infants with egg allergy, median age of diagnosis was 8.4 months (range 3.1–42 months), 43.4% were female, 90.4% developed well-baked egg tolerance, 58.5% outgrew egg allergy, 80.7% had atopic dermatitis (11.0% severe, 24.4% moderate and 45.1% mild), 67.1% had food allergies other than egg, 25.6% had asthma and 63.9% had parental allergic conditions. After adjusting for sex, children without atopic dermatitis were more likely to outgrow their egg allergy (adjusted OR 4.15, 95% CI 1.05–16.1, p = 0.042). Absent or mild atopic dermatitis was not associated with developing well-baked egg tolerance (6.45, 0.63–65.8, p = 0.077). Among children who remained allergic to well-baked egg, none had severe atopic dermatitis.

**Conclusions:** Children without atopic dermatitis were more likely to outgrow their egg allergy, although an association with atopic dermatitis severity could not be determined in this small study. Atopic dermatitis was not associated with development of tolerance to well-baked egg.

### #28 Prenatal diet and infant sensitization in the CHILD cohort

#### Keely Loewen^1^, Theo J. Moraes^2^, Stuart E. Turvey^3^, Piush J. Mandhane^4^, Malcom R. Sears^5^, Padmaja Subbarao^2^, Allan B. Becker^1^, Meghan B. Azad^1^, Elinor Simons^1^

##### ^1^Department of Pediatrics and Child Health and Children’s Hospital Research Institute of Manitoba - DEVOTION Network, University of Manitoba, Winnipeg, MB, Canada; ^2^Division of Respirology, Department of Pediatrics, Hospital for Sick Children, Toronto, ON, Canada; ^3^Division of Allergy and Immunology, Department of Pediatrics, University of British Columbia and British Columbia Children’s Hospital, Vancouver, BC, Canada; ^4^ Division of Pediatric Respirology, Pulmonary & Asthma, University of Alberta, Edmonton, AB, Canada; ^5^Division of Respirology, Department of Medicine, McMaster University, Hamilton, ON, Canada

**Correspondence:** Keely Loewen

*Allergy Asthma Clin Immunol* 2021, **17(Suppl 1):**28

**Background:** Early introduction of highly-allergenic foods has been associated with decreased risk of sensitization. We examined associations between maternal diet during pregnancy and child sensitization to egg, peanut, cow’s milk, and dust mites at ages 1 and 3 years.

**Methods:** CHILD participants were recruited from the general population before birth. Maternal diet, including number of days per week of egg consumption, was reported prenatally. Infant diet was reported at birth and every 3–6 months. At ages 1 and 3 years, sensitization to allergens, including egg, peanut, cow’s milk, and dust mites, was measured by skin prick testing. Atopic dermatitis was diagnosed clinically starting at age 6 months. Multivariable logistic regression was used to examine associations between maternal prenatal consumption of egg and other food allergens, timing of infant dietary introduction of food allergens, and child sensitization to egg, peanut, cow’s milk and dust mites.

**Results:** Among 2912 CHILD participants at age 1 year, sensitization was: 7.4% egg, 5.0% peanut, 15.5% cow’s milk, and 1.0% dust mites; at 3 years, sensitization was: 2.2% egg, 3.8% peanut, 1.1% cow’s milk, and 1.8% dust mites. After adjusting for potential confounders, such as moderate-severe atopic dermatitis, 1-year-old infants of mothers who ate egg at least daily during pregnancy (3.8%) were over twice as likely to be sensitized to egg (OR 2.63; 95%CI: 1.46–4.72), peanut (OR 2.48, 95% CI: 1.24–4.99) and cow’s milk (OR 2.34; 95%CI: 1.00–5.46), but not to dust mites. Results were similar for sensitization at age 3 years and persisted after stratification by age of introduction to the highly allergenic foods.

**Conclusions:** Infants were more likely to be sensitized to egg, peanut, and cow’s milk if their mothers ate egg daily during pregnancy. These associations persisted after accounting for age of highly-allergenic food introduction into the infants’ diets.

### #29 Biphasic Anaphylaxis: A prospective evaluation of incidence, risk factors, preventative features, and severity in a Canadian tertiary care centre

#### Leila A. Alenazy^1^, Grace Yin^2^, Hannah Botting^2^, Anne K. Ellis^3^

##### ^1^Department of Medicine, Queen’s University, Kingston, ON, Canada; ^2^Allergy Research Unit, Kingston Health Sciences Centre- KGH Site, Kingston, ON, Canada; ^3^Division of Allergy & Immunology, Department of Medicine, Queen’s University, Kingston, ON, Canada

**Correspondence:** Leila A. Alenazy

*Allergy Asthma Clin Immunol* 2021, **17(Suppl 1):**29

**Background:** Biphasic anaphylaxis is unpredictable and has wide range 1%–23% of incidences. Previous studies recognized delayed epinephrine administration as a risk factor for biphasic reactions. We have previously demonstrated an incidence rate of 19% for biphasic anaphylactic reactions in the Kingston. The purpose of this study is to determine the incidence of, and predictors for, biphasic anaphylaxis in a single centre via a prospective evaluation of patients with diagnosed anaphylaxis.

**Methods:** All patients with emergency department visits given a diagnosis of “allergic reaction”, “anaphylaxis”, “drug allergy”, or “insect sting allergy” during 1.5 year period were evaluated. Patients were contacted within 72 h to establish symptoms and determine the presence of biphasic reactivity. A full medical record review of the incident ensued, and uniphasic and biphasic cases were compared using the unpaired t test for continuous data and Fisher’s exact tests for ordinal data.

**Results:** A total of 155 patients with anaphylaxis were identified; complete follow-up was obtained for 138 patients. Twenty-two patients (16%) experienced confirmed biphasic reactivity, seven patients (5%) experienced secondary non-biphasic (SnBi) reactivity. Nineteen patients had their second phase occur > 8 h after the initial reaction; 63.6% were biphasic, and 71.4% were SnBi. There were no consistent clinical features or management differences predictive of biphasic reactors. The SnBi reactions were limited cutaneous manifestations.

**Conclusions:** Biphasic anaphylaxis incidence in this study was 16%. The second-phase onset can occur > 8 h after initial symptom resolution. Predicting biphasic anaphylaxis is still challenging as clinical presentations, and management were similar in this study.

### #30 “A Seedy Experience”: Understanding cross reactivity patterns in seed allergy

#### Vaidehi Bhatt, Jason A. Ohayon

##### Hamilton Allergy, Hamilton, ON, Canada

**Correspondence:** Vaidehi Bhatt

*Allergy Asthma Clin Immunol* 2021, **17(Suppl 1):**30

**Background:** Sesame seed is an emergent global food allergen with limited data on seed cross-reactivity [1]. Allergenic seed storage proteins such as 11S, 7S globulins, and 2S albumins found in tree nuts share homologous amino acid sequences in botanically-related plants and seeds [1], attributing to cross-reactivity. Sesame seed cross-reactivity is found in poppy seed [1]. Clinical cross-reactivity is also found between sesame and sunflower seeds shared by similar 2S albumin proteins [1]. Patients with single seed allergies often request information for risk to other seed allergies.

**Case Presentation:** A 31-year-old female patient with a history of allergy to tree nuts, sesame seed, and asthma, consumed a poppy seed cracker and developed throat hoarseness and gastrointestinal (GI) allergic symptoms. Treatment with epinephrine and antihistamine therapy resulted in prompt resolution of symptoms.

Skin prick test (SPT) identified positive responses to sesame seed, poppy seed, pumpkin seed, peanut, and tree nuts including almond, brazil nut, pecan, walnut, hazelnut, pistachio, and cashew. SPT was negative to sunflower seed and all indoor and outdoor inhalants.

**Conclusions:** The positive SPT responses to sesame, poppy, and pumpkin seeds suggest rare cross-reactivity among seed allergens. It is interesting to note, the negative SPT result to sunflower seed does not support observed cross-reactivity between sunflower and sesame seeds which both contain 2S albumin. Limited research regarding seed cross-reactivity is available but it is important to note that in vitro cross-reactivity may or may not reflect clinical cross-reactivity [1]. Testing and in-office oral challenge to seeds are necessary to clarify the extent of seed allergies in these patients.

**Reference**

1. Patel A, Bahna SL. Hypersensitivities to sesame and other common edible seeds. Allergy. 2016, 71(10):1405–13.

**Statement of Consent:** Written informed consent for this case report was obtained from the patient.

### #31 First real-world safety analysis of preschool sesame oral immunotherapy

#### Stephanie Erdle^1^, Lianne Soller^1^, Sandeep Kapur^2^, Gregory A. Rex^2^, Mary McHenry^2^, Victoria Cook^3^, Edmond S. Chan^1^

##### ^1^Division of Allergy and Immunology, Department of Pediatrics, University of British Columbia, Vancouver, BC, Canada; ^2^Division of Allergy, Department of Pediatrics, Dalhousie University/IWK Health Centre, Halifax, NS, Canada; ^3^Community Allergy Clinic, Victoria, BC, Canada

**Correspondence:** Stephanie Erdle

*Allergy Asthma Clin Immunol* 2021, **17(Suppl 1):**31

**Background:** In 2019, our group was the first to find preschool peanut oral immunotherapy (CPP-OIT) in a real-world multicenter setting safe for the vast majority of preschoolers. Our group sought to examine whether these findings could be replicated with sesame OIT (S-OIT), in the real-world setting.

**Methods:** As part of a Canada-wide quality improvement project S-OIT was administered to preschool-age children who had (1) skin prick test wheal diameter greater than or equal to 3 mm or specific IgE level greater than or equal to 0.35 kU/L and history of reaction and/or positive baseline oral food challenge, or (2) no ingestion history and specific IgE level greater than or equal to 5 kU/L. Over 9–11 visits patients had biweekly clinic visits for updosing and consumed the dose daily at home between visits. Target maintenance dose was 166 mg of sesame protein (1 mL tahini; 1 heaping teaspoon of hummus). This dose is approximately 10% of a full serving, which is comparable to the 7.5% of a full serving used for maintenance in CPP-OIT, so was chosen for ease of measuring. Symptoms were classified using a modified World Allergy Organization Subcutaneous Immunotherapy Reaction Grading System (1 mildest, 5 fatal).

**Results:** 9 patients started S-OIT in the period 2018–2019. The median age at S-OIT entry was 30 months, with a median SPT wheal diameter of 8 mm. All patients reached maintenance. 33.3% of patients experienced reactions during buildup: 11.1% grade 1 and 22.2% grade 2, with no grade 3-5 reactions, and no epinephrine administered.

**Conclusions:** We are the first group to describe preschool S-OIT in a real-world multicenter setting. This treatment appears to have an excellent safety profile in our preliminary analysis with only a third of patients having mild reactions, none of which were life-threatening or required epinephrine. Future work will include long-term safety and effectiveness.

### #32 Anaphylaxis to fruit from the Cross-Canada Anaphylaxis Registry (C-CARE)

#### Sofianne Gabrielli^1^, Ann E. Clarke^2^, Judy Morris^3^, Jocelyn Gravel^4^, Rodrick Lim^5^, Edmond S. Chan^6^, Ran D. Goldman^7^, Andrew O’Keefe^8^, Jennifer Gerdts^9^, Derek Chu^10^, Julia Upton^11^, Elana Hochstadter^12^, Adam Bretholz^13^, Christine McCusker^1^, Xun Zhang^14^, Moshe Ben-Shoshan^1^

##### ^1^Division of Allergy and Clinical Immunology, Department of Pediatrics, Montreal Children’s Hospital, McGill University Health Centre, Montreal, QC, Canada; ^2^Division of Rheumatology, Department of Medicine, Cumming School of Medicine, University of Calgary, Calgary, AB, Canada; ^3^Department of Emergency Medicine, Sacré-Coeur Hôpital, Montreal, QC, Canada; ^4^Division of Pediatric Emergency Medicine, Department of Pediatrics, Centre Hospitalier Universitaire Sainte-Justine,, Montreal, QC, Canada; ^5^Division of Pediatric Emergency Medicine, Department of Pediatrics, Children’s Hospital at London Health Science Centre, London, ON, Canada; ^6^Division of Allergy and Immunology, Department of Pediatrics, BC Children’s Hospital, University of British Columbia, Vancouver, BC, Canada; ^7^Division of Clinical Pharmacology and Emergency Medicine, Department of Pediatrics, BC Children’s Hospital, University of British Columbia, Vancouver, BC, Canada; ^8^Department of Pediatrics, Faculty of Medicine, Memorial University, St. John’s, NL, Canada; ^9^Executive Director, Food Allergy Canada, Toronto, ON, Canada; ^10^Division of Clinical Immunology & Allergy, Department of Medicine, and Department of Health Research Methods, Evidence & Impact, McMaster University, Hamilton, ON, Canada; ^11^Division of Immunology and Allergy, Department of Pediatrics, The Hospital for Sick Children, Department of Paediatrics, University of Toronto, Toronto, ON, Canada; ^12^Department od Pediatric Emergency Medicine, The Hospital for Sick Children, University of Toronto, Toronto, ON, Canada; ^13^Division of Pediatric Emergency Medicine, Department of Pediatrics, Montreal Children’s Hospital, Montreal, QC, Canada; ^14^Centre for Outcomes Research and Evaluation, Research Institute of McGill University Health Centre, Montreal, QC, Canada

**Correspondence:** Sofianne Gabrielli

*Allergy Asthma Clin Immunol* 2021, **17(Suppl 1):**32

**Background:** Data on fruit-induced anaphylaxis is sparse. We aimed to assess the clinical characteristics and management of patients presenting to Emergency Departments (ED) across Canada and to determine factors associated with severe reactions and epinephrine use.

**Methods:** Between April 2011 and May 2020, children and adults presenting with anaphylaxis to seven EDs in four Canadian provinces were recruited as part of the Cross-Canada Anaphylaxis Registry (C-CARE). A standardized form documenting symptoms, triggers, and management was collected. Multivariate logistic regression was used to identify factors associated with severe reactions and epinephrine treatment in the pre-hospital setting.

**Results:** Over a 9-year period, 248 patients with fruit-induced anaphylaxis presented to the EDs, median age was 10.2 [Interquartile Range (IQR) 3.7, 23.5] and 48.4% were males. The most common fruit triggers were kiwi (15.7%), banana (9.7%), and mango (9.7%). Only 23 patients (9.3%) reported having eczema. Epinephrine use was low in both the pre-hospital setting and the ED (28.6% and 40.7%, respectively), and 157 (63.3%) patients did not receive treatment with epinephrine at all.

Severe reactions to fruit were more likely to occur in spring and among those with known eczema [adjusted Odds Ratio (aOR) 1.12 (95% CI 1.03, 1.22 and 1.19 (95% CI 1.04, 1.37), respectively], while adjusting for age, sex, fruit trigger, known asthma, and epinephrine treatment. Patients with moderate and severe reactions [aOR 1.23 (95% CI 1.06, 1.44)] and those with a known food allergy [aOR 1.34 (95% CI 1.19, 1.50)] were more likely to be treated with epinephrine in the pre-hospital setting, while adjusting for age, sex, fruit trigger, known asthma, known eczema, and prescription of an epinephrine auto-injector.

**Conclusions:** Severe anaphylaxis to fruit could be due to cross-reactivity to pollens present in the spring. Epinephrine use in fruit-induced anaphylaxis is suboptimal therefore education programs prompting the use of epinephrine are needed.

### #33 Bridging knowledge gaps in anaphylaxis management through a video-based educational tool

#### Jumanah Karim^1^, Sofianne Gabrielli^1^, Bahar Torabi^1^, Adam Byrne^1^, Sarah De Schryver^1^, Vanessa Gadoury-Lévesque^1^, Chris McCusker^1^, Matthieu Vincent^1^, Judy Morris^2^, Jennifer Gerdts^3^, Xun Zhang^4^, Moshe Ben-Shoshan^1^

##### ^1^Division of Pediatric Allergy and Immunology, Department of Pediatrics, McGill University Health Centre, Montreal, QC, Canada; ^2^Department of Emergency Medicine, Sacré-Coeur Hôpital, Montreal, QC, Canada; ^3^Food Allergy Canada, Toronto, ON, Canada; ^4^Centre for Outcomes Research and Evaluation, Research Institute of McGill University Health Centre, Montreal, QC, Canada

**Correspondence:** Jumanah Karim

*Allergy Asthma Clin Immunol* 2021, **17(Suppl 1):**33

**Background:** Children with anaphylaxis are often not appropriately managed by caregivers. We aimed to develop and test the effectiveness of an education tool to help pediatric patients and their families better understand anaphylaxis and its management, and to improve current knowledge and treatment guidelines adherence.

**Methods:** From June 2019 to March 2020, 103 pediatric patients with history of food-triggered anaphylaxis who presented to the allergy outpatient clinics at the Montreal Children’s Hospital and The Children’s Clinic located in Montreal, Quebec were recruited. The patients and parents, together, were asked to complete six questions related to the triggers, recognition and management of anaphylaxis at the time of presentation to the clinic. Participants were automatically shown a 5-min animated video addressing the main knowledge gaps related to the causes and management of anaphylaxis. At the end of the video, participants were redirected to the same 6 questions to respond again. The scores were recorded in percentage of correct answers (minimum 0.0; maximum 1.0).

**Results:** The mean age of the patients was 5.4 ± 4.3 years (range: 0.6–17.9 years). The majority were males (54 patients; 52.4%). The mean baseline pre-video education questionnaire score was 0.77 ± 0.16 (range: 0.3–1.0), while the mean follow-up score was 0.82 ± 0.17 (range: 0.3–1.0). This score difference of 0.05 was statistically significant (p = 0.001). There were no significant associations between change in scores and age or sex of the participants.

**Conclusions:** Our video teaching method was successful in educating patients and their families to better understand anaphylaxis and its management at the moment of the clinical encounter. The test will be repeated at a 1-year interval to determine their retention of knowledge. Other educational methods should be developed and investigated to improve the knowledge of participants with worse (or no change) follow-up scores in our study.

**Acknowledgement:** This project received support from AllerGen, the Allergy, Genes and Environment Network, which is a national research network funded by Innovation, Science and Economic Development Canada through the Networks of Centres of Excellence (NCE) Program.

### #34 Reproducibility of symptom sequences across episodes in patients with recurrent anaphylaxis

#### Calum Slapnicar^1^, Gerald Lebovic^1^, Matthew Dozois^2^, Aidan McParland^2^, Peter Vadas^1^

##### ^1^St. Michael’s Hospital, Toronto, ON, Canada; ^2^University of Toronto Faculty of Medicine, Toronto, ON, Canada

**Correspondence:** Calum Slapnicar

*Allergy Asthma Clin Immunol* 2021, **17(Suppl 1):**34

**Background:** This study sought to examine the temporal sequence of manifestations of anaphylaxis within individuals and between individuals across multiple reactions.

**Methods:** Patients evaluated for recurrent anaphylaxis in a tertiary care allergy clinic between 2012–2018 were included. At each visit, patients were asked to record the temporal sequence in which symptoms of anaphylaxis appeared. The Fleiss’ Kappa method was used to assess reproducibility of the order of appearance of specific symptoms during anaphylaxis in individual patients and across individuals with similar triggers.

**Results:** Mean patient age was 35.7 years (SD = 14.0; range 1–71) of whom 77% were female. A total of 3129 anaphylactic reactions were analyzed in 159 patients. The mean kappa within individuals was 0.93, 5th percentile was 0.49 and 95th percentile was 1.0. The mean kappa between individuals of the same allergic trigger was − 0.029 [range, 0.11–0.05]. Among the 19 patients who recorded reactions of varying severity levels, the mean within-patient kappa was 0.94, with 15/19 patients having values of 1.0.

**Conclusions:** These data suggest that patients will continue to experience reproducibly stereotypic anaphylactic reactions over time. Furthermore, when reactions escalate in severity, the sequence of early symptoms remains unchanged. The sequence of symptoms of anaphylaxis is unique to a given individual, but not across individuals who share a common allergic trigger. By recognizing the stereotypic nature of anaphylactic reactions, especially the earliest symptoms, patients will be better able to identify incipient reactions and intervene appropriately with self-administration of epinephrine and by activating emergency medical services.

### #35 An unusual exposure to cow milk triggering an allergic reaction in an infant

#### Wade T. Watson^1,2^

##### ^1^Dalhousie University, Halifax, NS, Canada; ^2^IWK Health Centre, Halifax, NS, Canada

**Correspondence:** Wade T. Watson

*Allergy Asthma Clin Immunol* 2021, **17(Suppl 1):**35

**Background:** For many children, exposure to foods to which they are allergic commonly occur through accidental exposure or food contamination. We present a case of an infant with cow milk allergy exposed to partially digested cow milk in vomitus who experienced a significant allergic reaction.

**Case Presentation:** A 1 year old male was diagnosed at age 6 months with allergies to cow milk, egg and possibly mustard. He attends a day care with five other infants in the same room. Another infant was fed cow milk from a bottle. Approximately 2 h and 15 min later that infant vomited close to the subject. He was exposed to the vomitus on his clothes, hands and face. He was cleaned immediately but urticaria were noted on areas where the vomitus had touched his skin. Within minutes he started to drool and he began to cough. He was given i.m. epinephrine (EpiPen^®^) and transported to the local emergency department. The cough and drooling rapidly improved. On arrival to the emergency department there were no other symptoms and his vital signs were normal. He was observed there for 4 h. The specific IgE level (ImmunoCAP^®^) to cow milk was subsequently measured and was greater than 100 kU/L.

**Conclusions:** This was an unusual exposure to cow milk. Even after 2 h in an infant’s stomach, the cow milk allergens had not completely digested and were still capable of triggering an allergic reaction. This reinforces the need for close supervision of infants and young children with food allergies and immediately cleaning food waste, including vomitus, from surfaces to prevent accidental exposure.

**Statement of Consent:** Written informed consent for this case report was obtained from the patient.

### #36 Elucidating mechanisms that maintain early allergic memory in food allergy

#### Youssef El-Sayes^1^, Emily Grydziuszko^1^, Maggie Chopra^1^, Joshua Koenig^1^, Roopali Chaudhary^1^, Rodrigo Jimenez-Saiz^1^, Kelly Bruton^1^, Tina Walker^1^, Malcolm Davidson^1^, Owen Baribeau^1^, Jianping Wen^1^, Saba Manzoor^1^, Siyon Gadkar^1^, Allyssa Phelps^1^, Justin Taylor^2^, Susan Waserman^1^, Manel Jordana^1^

##### ^1^McMaster University, Hamilton, ON, Canada; ^2^Fred Hutchinson Cancer Research Center, Seattle, WA, USA; ^3^University of Washington, Seattle, WA, USA

**Correspondence:** Youssef El-Sayes

*Allergy Asthma Clin Immunol* 2021, **17(Suppl 1):**36

**Background:** Food allergies are mediated by allergen-specific (as) –IgE which binds to the surface of mast cells and basophils, and when bound by allergen, causes these cells to release inflammatory mediators that result in allergic symptoms.

A recent randomized trial found that allergen exposure in the first 6 months of life reduced the likelihood of allergic sensitization compared to later exposure [1], suggesting that sensitization events in allergic patients may occur very early in life.

However, the transition from early sensitization to clinical manifestation of allergic responses is a critical phenomenon that requires further elucidation. Therefore, our objective is to characterize the early sensitization phase and the mechanisms that lead to overt allergy.

**Methods:** Our novel murine model illustrates that a single intragastric administration of an allergen that in itself is not associated with the generation of IgE, does establish lifelong immunological memory such that subsequent allergen exposures lead to the production of IgE and anaphylaxis.

**Results:** Using a novel model of allergic priming to investigate the early, incipient presentation of food allergy, we found that the memory phenotype imprinted during priming is long-lived and potentially permanent.

T cell proliferation in vitro and functional measures of T cell activity suggest the presence of activated memory Th2 cells. Additionally, allergen-specific B cells seem naive in secondary lymphoid tissues post-priming.

**Conclusions:** Overall, we propose that the trajectory of allergic immunity starts with a pre-clinical, activated T cell phase which is insufficient to activate B cells. These T cells hold the memory of IgE responses. Upon secondary exposure, these T cells show a capacity to interact with naive B cells and facilitate differentiation and IgE class switching.

**References**

1. Du Toit G, Roberts G, Sayre PH, Bahnson HT, Radulovic S, et al. Randomized trial of peanut consumption in infants at risk for peanut allergy. N Engl J Med. 2015;372(9):803–13.

### #37 Clinical predictors of food allergy in a Canadian allergy clinic

#### Joella Ho^1^, Adam Aue^2^, Harold Kim^3, 4^, Samira Jeimy^4^

##### ^1^Schulich School of Medicine and Dentistry, Western University, London, ON, Canada; ^2^Faculty of Medicine, University of Ottawa, Ottawa, ON, Canada; ^3^Department of Medicine, McMaster University, Hamilton, ON, Canada; ^4^Division of Clinical Immunology and Allergy, Department of Medicine, Western University, London, ON, Canada

**Correspondence:** Joella Ho

*Allergy Asthma Clin Immunol* 2021, **17(Suppl 1):**37

**Background:** The pathogenesis of food allergy remains unclear, though several theories have been proposed. Determining predictors for food allergy may guide investigations of its pathogenesis and help to detect patients at risk before a serious life-threatening reaction occurs. This study aimed to identify predictors of food allergy from a patient’s clinical history.

**Methods:** A retrospective chart review of patients referred for assessment of food allergy at a Canadian hospital-based allergy clinic was conducted. Patients were considered to have food allergy based on unsuccessful oral food challenge or clinical judgment. Complete-case analysis was conducted. Patients missing data and those for whom a diagnosis of food allergy could not be confirmed or ruled out were excluded from further analysis. Logistic regression was used to determine factors that predict food allergy.

**Results:** 41 (72%) of referred patients were ultimately diagnosed with food allergy. In our patient population, univariate analysis identified that the following factors were associated with a food allergy: family history of atopy (Odds Ratio (OR) = 2.85, 95% Confidence Interval (CI) = 1.51, 5.35), asthma (OR = 2.86, 95% CI = 1.21, 6.76), eczema (OR = 3.71, 95% CI = 1.61, 8.56), allergic rhinitis (OR = 3.25, 95% CI = 1.47, 7.18), having a pet at home (OR = 3.5, 95% CI = 1.59, 7.68) and male sex (OR = 2.67, 95% CI = 1.24, 5.74). Multivariate analysis identified age over 18 as a negative predictor (OR = 0.10, 95% CI = 0.012, 0.72).

**Conclusions:** Personal and family history of atopy increase risk of food allergy, supporting the findings of previous studies. The factors identified in this study may be intercorrelated, requiring a larger sample size to separate their effects. Other factors not investigated may also play a greater role in predicting food allergy, highlighting the need for further research to identify and quantify predictors of allergic reactions.

### #38 Multivariate analysis of factors influencing oral food challenge decision-making

#### Joella Ho^1^, Adam Aue^2^, Harold Kim^3, 4^, Samira Jeimy^4^

##### ^1^Schulich School of Medicine, Western University, London, ON, Canada; ^2^Faculty of Medicine, University of Ottawa, Ottawa, ON, Canada; ^3^ Department of Medicine, McMaster University, Hamilton, ON, Canada; ^4^Division of Clinical Immunology and Allergy, Department of Medicine, Western University, London, ON, Canada

**Correspondence:** Joella Ho

*Allergy Asthma Clin Immunol* 2021, **17(Suppl 1):**38

**Background:** Oral food challenges (OFCs) involve the ingestion of a suspected food allergen under medical supervision and are the diagnostic standard of food allergies. OFCs are recommended for patients below positive decision points, where there is a 95% probability of a reaction. However, global consensus on approach is lacking. This study investigated OFC use in an allergy clinic and sought to identify clinical factors that influence the decision to pursue OFC.

**Methods:** A retrospective chart review of patients referred for investigation of food allergy at a hospital-based, Canadian allergy clinic was conducted. Patients with missing data and those ultimately diagnosed with eosinophilic esophagitis, protein intolerance, celiac disease, and food protein induced proctocolitis/enterocolitis were excluded from further analysis. Clinical factors associated with OFC decision-making were modelled using multivariate logistic regression.

**Results:** Out of 71 cases presenting with a query of food allergy, 15 (21%) completed OFC, while 56 (79%) did not complete OFC. Of patients tested with OFC, 9 (60%) passed OFC and were determined not to have food allergy. No patients experienced an anaphylactic reaction above a grade 3 on the World Allergy Organization systemic allergic reaction grading system. Factors associated with the decision to pursue OFC were skin prick test wheal size (Odds Ratio (OR) = 0.78, 95% Confidence Interval (CI) = 0.65, 0.94), age at index reaction (OR = 0.89, 95% CI = 0.80, 0.99), and the report of oral symptoms in the index reaction (OR = 6.69, 95% CI = 1.28, 34.88).

**Conclusions:** In this study, OFCs were used in approximately 1 in 5 investigations of food allergy. Age and oral symptoms at index reaction and skin prick test wheal size predict OFC use but may not adequately predict risk for anaphylaxis during OFC. Further research is needed to identify predictors of likelihood and severity of allergic reactions and provide consensus in the use of OFCs.

### #39 Tree nut-induced anaphylaxis in Canadian emergency departments: rate, clinical characteristics and management

#### Laurence Ducharme^1^, Sofianne Gabrielli^1^, Ann E. Clarke^2^, Judy Morris^3^, Jocelyn Gravel^4^, Rodrick Lim^5^, Edmond S. Chan^6^, Ran D. Goldman^7^, Andrew O’Keefe^8^, Jennifer Gerdts^9^, Derek K. Chu^10^, Julia Upton^11^, Elana Hochstadter^11^, Adam Bretholz^12^, Christine McCusker^1^, Xun Zhang^13^, Moshe Ben-Shoshan^1^

##### ^1^Division of Allergy and Clinical Immunology, Department of Pediatrics, Montreal Children’s Hospital, McGill University Health Centre, Montreal, QC, Canada; ^2^Division of Rheumatology, Department of Medicine, University of Calgary, Canada, AB, Canada; ^3^Department of Emergency Medicine, Sacré-Coeur Hôpital, Montreal, QC, Canada; ^4^Division of Pediatric Emergency Medicine, Department of Pediatrics, Centre Hospitalier Universitaire Sainte-Justine, Montreal, QC, Canada; ^5^Division of Pediatric Emergency Medicine, Department of Pediatrics, Children’s Hospital at London Health Science Centre, London, ON, Canada; ^6^Division of Allergy and Immunology, Department of Pediatrics, BC Children’s Hospital, University of British Columbia, Vancouver, BC, Canada; ^7^Division of Clinical Pharmacology and Emergency Medicine, Department of Pediatrics, BC Children’s Hospital, University of British Columbia, Vancouver, BC, Canada; ^8^Division of Allergy and Immunology, Department of Pediatrics, Faculty of Medicine, Memorial University, St. John’s, NL, Canada; ^9^Executive Director, Food Allergy Canada, Toronto, ON, Canada; ^10^Division of Clinical Immunology & Allergy, Department of Medicine, and Department of Health Research Methods, Evidence & Impact, McMaster University, Hamilton, ON, Canada; ^11^Division of Immunology and Allergy, Department of Pediatrics, The Hospital for Sick Children, Department of Paediatrics, University of Toronto, Toronto, ON, Canada; ^12^Division of Pediatric Emergency Medicine, Department of Pediatrics, Montreal Children’s Hospital, Montreal, QC, Canada; ^13^Centre for Outcomes Research and Evaluation, Research Institute of McGill University Health Centre, Montreal, QC, Canada

**Correspondence:** Laurence Ducharme

*Allergy Asthma Clin Immunol* 2021, **17(Suppl 1):**39

**Background:** Data on rate, clinical characteristics and management of tree nut-induced anaphylaxis in Canada is sparse. We aimed to characterize the rate, clinical characteristics and management of tree nut-induced anaphylaxis in children (0–17 years old) across Canada, to compare West and East coasts, and to assess factors associated with severe reactions (defined as including stridor, cyanosis, circulatory collapse, or hypoxia).

**Methods:** Between 2011 and 2020, children presenting to five emergency departments (EDs) were recruited as part of the Cross-Canada Anaphylaxis REgistry (C-CARE). A standardized form documenting symptoms, triggers and management were collected. The leading culprits, symptoms and epinephrine use between West coast (one center in British Columbia) and East coast (three centers in Quebec, one center in Ontario) EDs were compared. Multivariate logistic analysis was used to evaluate factors associated with severe reactions.

**Results:** A total of 1310 cases of anaphylaxis were included, among which 540 (41%) were induced by tree nuts. The median age was 5.20 years [Interquartile range (IQR): 2.50–9.50] and 65.4% were males. Among all reactions, 7.0% were severe. The major tree nuts accounting for anaphylaxis were cashew, hazelnut and pistachio (32.8%, 20.0% and 9.3%, respectively). Cashew-induced anaphylaxis was more common in the West coast [14.0% difference (95% CI, 1.6%–27.6%)], whereas pistachio-induced anaphylaxis was more common in the East coast [6.3% difference (95% CI, 0.5%–12.2%)].

Pre-hospital and ED epinephrine administration was documented in 35.2% and 52.5% of cases, respectively. Severe reactions were more likely among males [adjusted Odds Ratio (aOR) 1.05 (95% CI, 1.01–1.10)] and among older children [aOR 1.00 (95% CI, 1.00–1.01)], while adjusting for location and type of tree nut.

**Conclusions:** Different tree nut-induced anaphylaxis patterns within Canada may be due to differences in lifestyle. Educational programs promoting prompt epinephrine use and vigilance regarding the risk of severe reactions are required, especially among males and older children.

## Immunology

### #40 An unusual presentation of ADA2 deficiency

#### Lydia Zhang, Reza Alizadehfar

##### McGill University Health Centre, Montreal, QC, Canada

**Correspondence:** Lydia Zhang

*Allergy Asthma Clin Immunol* 2021, **17(Suppl 1):**40

**Background:** Deficiency of adenosine deaminase type 2 (DADA2) resulting from recessive loss-of-function mutations in *ADA2* was identified in 2014 and described as new auto-inflammatory syndrome presenting with recurrent fevers, early-onset stroke, systemic vasculitis and mild immunodeficiency. Our understanding of this pathology has since expanded. A genotype–phenotype spectrum in DADA2 was recently identified with three distinct presentations depending on the ADA2 mutation: vasculitis, pure red cell aplasia and bone marrow failure. We present a case of DADA2 presenting with bone marrow failure 25 years after diagnosis of common variable immune deficiency (CVID).

**Case Presentation:** Our patient is a 33-year-old gentleman coming from a non-consanguineous French-Canadian family. After a diagnosis of immune thrombocytopenia at age 3 and several respiratory tract infections, he was evaluated in immunology at age 6. Work-up revealed panhypogammaglobulinemia and suboptimal vaccine responses. Complete blood count, lymphocyte subset enumeration as well as T cell proliferation to mitogens were normal. CVID was diagnosed and he was started on immunoglobulin replacement therapy without further significant infections. Abdominal ultrasounds revealed a gradual increase in splenomegaly, with thrombocytopenia noted at age 27. Abdominal imaging at age 31 revealed a 30 cm spleen, non-cirrhotic portal hypertension with esophageal varices as well as axillary and inguinal lymphadenopathies. A homozygous pathogenic missense mutation in ADA2 c.1072G>A (p.Gly358Arg) was detected on genetic panel testing. Subsequent bone marrow biopsy revealed myelofibrosis. The patient was started on adalimumab with consideration for future bone marrow transplant.

**Conclusions:** ADA2 is likely involved in bone marrow microenvironment but its physiologic functions are not fully understood. DADA2 can present clinical overlap with CVID, but whether CVID is a distinct phenotype in DADA2 remains to be clarified.

**Statement of Consent:** Written informed consent for this case report was obtained from the patient.

### #41 Inborn errors of immunity associated with type 2 inflammation in the USIDNet registry

#### Kelsey L. Smith^1,2^, Bhavi Modi^1,2^, Darlene Dai^1,2^, Elizabeth Secord^3,4^, Kathleen Sullivan^5,6^, Elizabeth Garabedian^7,8^, Stuart E. Turvey^1,2^, Catherine M. Biggs^1,2^

##### ^1^University of British Columbia, Vancouver, BC, Canada; ^2^ BC Children’s Hospital, Vancouver, BC, Canada; ^3^Children’s Hospital of Michigan, Detroit, MI, USA; ^4^Wayne State University, Detroit, MI, USA; ^5^The Children’s Hospital of Philadelphia, Philadelphia, PA, USA; ^6^The University of Pennsylvania Perelman School of Medicine, Philadelphia, PA, USA; ^7^National Human Genome Research Institute, Bethesda, MD, USA; ^8^National Institutes of Health, Bethesda, MD, USA

**Correspondence:** Kelsey L. Smith

*Allergy Asthma Clin Immunol* 2021, **17(Suppl 1):**41

**Background:** Inborn errors of immunity (IEI) are a group of heritable monogenic conditions in which immune system development is interrupted or malfunctions. Allergic disease is an increasingly recognized feature of IEI, however, few studies have evaluated which IEIs are associated with type 2 inflammation. We looked for laboratory evidence of type 2 inflammation in IEIs using the United States Immunodeficiency Network (USIDNet) registry data.

**Methods:** A query was submitted to the USIDNet requesting clinical, molecular and laboratory data for a cohort of patients with a diagnosed IEI. Thresholds for elevated eosinophil (500 cell/uL) and IgE (137 IU/ml) levels were established based on literature references. Eosinophil and IgE levels were measured in 732 and 463 patients, respectively, for whom pre-transplant levels and genetic diagnoses were available. Proportion of eosinophilia and elevated IgE were compared to literature reference populations, respectively, using a 2 proportion z-test.

**Results:** Laboratory evidence of type 2 inflammation was common in IEI patients: 22% of those evaluated had eosinophilia and 63% had elevated IgE levels, compared to < 5% and 10% of literature reference populations, respectively. There were 15 unique IEI genes significantly associated with eosinophilia (*BTK, CTLA4, PIK3CD, FOXP3, TNFRSR13B, MAGT1, RAG1, NLRP3, IFNGR1, ADA, NFKB2, CYBB, DOCK8, WAS STAT3*), and 13 significantly associated with elevated IgE (*CYBA, IFNGR1, NCF1, FOXP3, NCF2, ITGB2, CTL4, ADA, WAS, AIRE, CYBB, DOCK8, STAT3*).

**Conclusions:** Eosinophilia and elevated IgE levels are common in patients with IEIs. In addition to recognizing established genes associated with allergic inflammation, this study identified IEIs not previously associated with type 2 inflammation.

**Statement of Consent:** Written informed consent for this case report was obtained from the patient.

### #42 Development of autoimmune hepatitis in a patient with common variable immunodeficiency on immunoglobulin replacement therapy

#### Uliana Kovaltchouk, Chrystyna Kalicinsky

##### University of Manitoba, Winnipeg, MB, Canada

**Correspondence:** Uliana Kovaltchouk

*Allergy Asthma Clin Immunol* 2021, **17(Suppl 1):**42

**Background:** Common variable immunodeficiency (CVID) is characterized by reduced serum immunoglobulins, and deregulation of the immune system. CVID has been associated with an increased incidence of some forms of cancers and autoimmune conditions.

**Case Presentation:** We describe a patient, diagnosed with CVID in her fourth decade of life, who subsequently developed autoimmune hepatitis after 2 years of immunoglobulin replacement therapy. Histological, and serological analyses were in keeping with the diagnosis of autoimmune hepatitis. She was initiated on azathioprine 75 mg orally once daily, with normalization in liver biochemistry and synthetic function.

**Conclusions:** This case suggests that although immunoglobulin replacement alleviates the general state of chronic immune activation, not all abnormalities in cellular immunity are normalized. There are few reported cases of autoimmune hepatitis associated with CVID in the current literature; there remains no specific therapy for liver involvement in CVID. We demonstrate clinical and biochemical control of autoimmune hepatitis with azathioprine in conjunction with continued immunoglobulin replacement therapy. Further research is needed to better understand what is driving immune activation, and to further characterize why patients still suffer from inflammatory complications even when on replacement therapy.

Written informed consent for publication of clinical details was obtained from the patient. A copy of the consent is available for review by the Editor of this journal.

### #43 Ontario Immunoglobulin Treatment (ONIT): A multi-centre program and clinical case registry to evaluate and standardize management for immunodeficiency

#### Juthaporn Cowan^1^, Stephen Betschel^2^, Susan Waserman^3^, Antonio Giulivi^4^, D. William Cameron^1^

##### ^1^Department of Medicine, University of Ottawa at the Ottawa Hospital, Ottawa, ON, Canada; ^2^Department of Medicine, University of Toronto, Toronto, ON, Canada; ^3^Department of Medicine, McMaster University, Hamilton, ON, Canada; ^4^Department of Pathology and Laboratory Medicine, University of Ottawa, Ottawa, ON, Canada

**Correspondence:** Juthaporn Cowan

*Allergy Asthma Clin Immunol* 2021, **17(Suppl 1):**43

**Background:** Healthcare for immunodeficiency has been fragmented, resulting in no uniform standard for diagnosis and management. A mainstay treatment, Immunoglobulin replacement therapy (IGRT) reduces infection, improve quality of life and survival. Cases of immunodeficiency, IG indications and utilization are increasing. Home-based, self-administered subcutaneous immunoglobulin (SCIG) has better patient satisfaction and decreased costs versus in-centre IVIG. However, evaluation of diagnosis, indication, adherence, dose and titration, therapeutic and adverse outcomes requires real-time population-based data, and has generally not been done.

**Methods:** We began a pilot multicentre clinical program dedicated to immunodeficiencies and IGRT. A key component of the clinic-based program is case management with specialized nursing care to educate, coordinate, support and monitor IGRT. We created a consented case registry using the ONIT infrastructure to register clinical and demographic data prospectively from the point of care. Clinical data include diagnosis, comorbidities, therapeutic indication for IGRT, specific treatment regimen, health outcomes, infection rates, adverse events and health-related quality of life to name a few.

**Results:** ONIT was approved by the Ontario MOHLTC in 2019 to promote and support SCIG, and evaluation. We have started at The Ottawa Hospital, St. Michael’s Hospital, and Hamilton Health Sciences. Recruitment of program nurses (4.0 FTE) was completed in March 2020. As of March 2020, we identified 564 SCIG and 118 IVIG patients in our program (uncurated data, before ONIT case registry). Case Report Forms were created and agreed upon, the case registry was approved by Clinical Trials Ontario (#1978), and a web-based case registry was successfully created by the Ottawa Methods Centre. The first patient was consented and enrolled in June 2020.

**Conclusions:** This pilot initiative is intended to generate high-quality data that can be used for healthcare evaluation, standardization and research for immunodeficient patients, which may improve IG stewardship and be used in other centres.

### #44 Primary immunodeficiency: increasing the diagnostic yield

#### Joseph Jacher^1^, Päivi Kokkonen^2^, Anni Niskakoski^2^, Inka Saarinen^2^, Johanna Sistonen^2^, Heidi Junnila^2^, Annakarin Kere^2^, Margarita Andreeskaya^2^, Mikko Muona^2^, Janica Djupsjöbacka^2^, Lotta Koskinen^2^, Hatice Duzkale^2^, Julie Hathaway^1^, Samuel Myllykangas^2^, Juha Koskenvuo^2^, Tero-Pekka Alastalo^1^

##### ^1^Blueprint Genetics, Seattle, WA, USA; ^2^Blueprint Genetics, Helsinki, Finland

**Correspondence:** Joseph Jacher

*Allergy Asthma Clin Immunol* 2021, **17(Suppl 1):**44

**Background:** Identifying the genetic etiology in patients with primary immunodeficiency (PID) is becoming increasingly important, both for patient prognosis, management and for estimating the risk for relatives. The diagnostic yield in this patient cohort varies between 15–79% depending on multiple factors including methodological differences such as platform used, the number of genes included and the risk for a monogenetic cause of disease. We report our experience with 2162 patients suspected to have a genetic cause of their PID. We describe the supplementary methods used for genes that cannot be reliably analysed by next-generation sequencing (NGS).

**Methods:** We performed a retrospective review of the NGS results of 2162 patients referred to our laboratory with a clinical suspicion of a PID and tested with one of our 10 immunology-related panels.

**Results:** The cumulative diagnostic yield of all immunology-related panels was 14.4% (311/2162). The diagnostic yield in patients between 0–5 years of age was 19.9%. Phenotypic-specific panels, like the Severe Combined Immunodeficiency Panel and the Chronic Granulomatous Disease had a higher diagnostic yield (47.4% and 46%, respectively) than the comprehensive Primary Immunodeficiency Panel (12.8%).

Of the 270 reported pathogenic and likely pathogenic diagnostic sequence variants, 217 (80%) were unique. Thirty diagnostic copy number variants (CNVs) were reported in 29 patients. In 3 index cases, our CNV detection algorithm identified a homozygous whole *NCF1* gene deletion. Because the analysis of *NCF1* is complicated by two highly homologous pseudogenes, additional bioinformatic analysis and a custom Sanger assay was performed to confirm these findings.

**Conclusions:** This study provides further evidence about the diagnostic yield of panel genetic testing in patients with PID. Younger patients and phenotype-specific panels have a higher diagnostic yield. Additional methods are needed to identify variants in difficult to sequence regions.

### #45 Real-world CANadian Cuvitru Noninterventional study in subjects transitioning from subcutaneous Immunoglobulin (CANCUN)

#### Paul K. Keith^1^, Jason Lee^2^, Juthaporn Cowan^3^, Amin Kanani^4^, Gina Lacuesta^5^, André Gladiator^6^, Claudia Schwarz^7^, Michelle Park^8^, Seema Agrawal^8^, Harold Kim^1, 9^

##### ^1^Division of Clinical Immunology and Allergy, Department of Medicine, McMaster University, Hamilton, ON, Canada; ^2^Division of Rheumatology, Mount Sinai Hospital, University Health Network, Toronto, ON, Canada; ^3^Division of Infectious Diseases, Department of Medicine, Department of Biochemistry, Microbiology, and Immunology Centre for Infection, Immunity and Inflammation (CI3), University of Ottawa, Ottawa, ON, Canada; ^4^Division of Allergy and Clinical Immunology, St. Paul’s Hospital, Department of Medicine, University of British Columbia, Vancouver, BC, Canada; ^5^Department of Medicine, Dalhousie University, Halifax, NS, Canada; ^6^Shire International GmbH, a Takeda compan, Zug, Switzerland; ^7^Baxalta Innovations GmbH, a Takeda company, Vienna, Austria; ^8^Shire US Inc., a Takeda company, Cambridge, MA, USA; ^9^Division of Clinical Immunology and Allergy, Department of Medicine, Western University, London, ON, Canada

**Correspondence:** Paul K. Keith

*Allergy Asthma Clin Immunol* 2021, **17(Suppl 1):**45

**Background:** Immune Globulin Subcutaneous (Human) 20% (Cuvitru; Ig20Gly) is a subcutaneous immunoglobulin (SCIG) formulation allowing for infusions at increased rates and reduced times compared with other conventional SCIG therapies. Real-world data on Ig20Gly use in Canada are limited. CANCUN (NCT03716700) is assessing real-world Ig20Gly infusion parameters in patients with primary and secondary immunodeficiencies (PID and SID, respectively) transitioning to Ig20Gly.

**Methods:** Patients (> 2 y) with PID or SID receiving SCIG for ≥ 3 months before transitioning to Ig20Gly were eligible for this ongoing Canadian multicenter phase 4, noninterventional, prospective, single-arm study. A prespecified interim analysis assessed baseline characteristics and Ig20Gly utilization at Ig20Gly initiation and 3, 6, and 12 months later. Safety data will be evaluated at final analysis.

**Results:** As of December 31, 2019, 121 patients were enrolled (64.5% female; 51.2% aged ≥ 65 y [mean; range: 61.9; 19–83 y]) at 6 centers. 57 (47.1%) patients had PID, 63 (52.1%) had SID (of whom 57.8% had chronic lymphocytic leukemia), and one had both. Most patients used 2 infusion sites (range: 1–6 sites; primarily lower and upper abdomen); mean site number (2.5) remained unchanged 3 months postinitiation. The mean maximal infusion rate was 28.8 mL/h/site at initiation but ≥ 48.3 mL/h/site at all postinitiation timepoints. The mean infusion duration was < 1 h at all timepoints (range: 5–135 min). The median dose was 8 g at all timepoints except 3 months (6.5 g). Most patients (> 70%) administered treatment once weekly. Interrupted or slowed infusions were rare (one at 6 months; two at 12 months).

**Conclusions:** In patients with PID or SID transitioning to Ig20Gly, infusions were more rapid after initiation, requiring < 1 h on average. Most commonly, patients used 2 infusion sites and administered Ig20Gly once weekly.

Shire US Inc. (a Takeda company) funded this study. Baxalta US Inc. (a Takeda company) funded writing support.

### #46 An atypical case presentation of X-linked Lymphoproliferative Disease Type 1 (XLP1) in adulthood

#### Daniel H. Li^1^, Gordon Sussman^2^, Raymond H. Kim^3^, Christine Song^4^

##### ^1^Department of Internal Medicine, University of Toronto, Toronto, ON, Canada; ^2^University of Toronto, Toronto, ON, Canada; ^3^Department of Medical Oncology, University Health Network, Toronto, ON, Canada; ^4^Department of Allergy and Immunology, St. Michael’s Hospital, Toronto, ON, Canada

**Correspondence:** Daniel H. Li

*Allergy Asthma Clin Immunol* 2021, **17(Suppl 1):**46

**Background:** X-linked lymphoproliferative disease type 1 (XLP1) is a very rare condition that commonly manifests as: hypogammaglobulinemia, lymphoma and hemophagocytic lymphohistiocytosis (HLH). Patients commonly present prior to the age of 10 and survival is poor without hematopoietic stem cell transplantation (HSCT). Here we present a rare case of an adult patient presenting with hypogammaglobulinemia diagnosed with XLP1.

**Case Presentation:** A 34-year-old man was referred for evaluation of hypogammaglobulinemia. He was found to have recurrent sinopulmonary infections and otitis media since infancy. He also had a history of Burkitt lymphoma diagnosed and treated at the age of 15 and has since been in remission. Baseline immunologic testing revealed evidence of hypogammaglobulinemia with a total IgG of 1.95 g/l, IgA of 0.179 g/l and IgM of 0.18 g/l. Absolute CD19 count was slightly low at 0.061 × 10^9^/L. A thorough family history revealed a pedigree suggestive of an X-linked disorder, prompting genetic testing. Molecular genetic testing found a likely pathogenic mutation in SH2D1A leading to a diagnosis of XLP1. In addition to his treatment with subcutaneous immunoglobulin replacement therapy he was also referred to a lymphoma specialist, due to concerns for lymphoma recurrence and HLH.

**Conclusions:** This case highlights the importance of a detailed family history in the assessment of adult patients with hypogammaglobulinemia. XLP1 is associated with unique risks and management considerations when compared with most other primary humoral immunodeficiencies diagnosed in adults, such as common variable immunodeficiency.

**Statement of Consent:** Written informed consent for this case report was obtained from the patient.

### #47 The Impact of Primary Immunodeficiency Informed Molecular Landscape on the Biology and Prognosis of Refractory Childhood and Adult Malignancies

#### Son Tran^1^, Luis Murguia-Favela^2^, Aru Narendran^1^

##### ^1^Division of Pediatrics, Section of Pediatric Oncology, Alberta Children’s Hospital and University of Calgary, Calgary, AB, Canada; ^2^Division of Pediatrics, Section of Pediatric Hematology and Immunology, Alberta Children’s Hospital and University of Calgary, Calgary, AB, Canada

**Correspondence:** Son Tran

*Allergy Asthma Clin Immunol* 2021, **17(Suppl 1):**47

**Background:** Cancer is one of the most common causes of death in patients with primary immunodeficiencies (PIDs). The immune surveillance concept postulates that PIDs are an important contributor to cancer growth and outcomes, as the integrity of the immune system is critical in the surveillance of abnormal cellular transformations and cancer cell survival. Although several immunodeficiency syndromes have been known to be associated with malignancies in children and adults, currently, the molecular mechanisms that link immune functions to cancers are poorly understood. We identified distinct molecular characteristics in cancers with respect to PID-related genes that impact survival outcomes in affected patients.

**Methods:** In this study, we integrated transcriptomic and somatic mutation data of 29 adult cancers and 5 pediatric cancers from publicly available databases such as The Cancer Genome Atlas (TCGA) and TARGET with respective healthy tissues as control from the Genotype-Tissue Expression (GTEx) project. PID-related genes were curated from the Fulgent Genetics “Comprehensive Primary Immunodeficiency NGS Panel.” Unified datasets integrating GTEx healthy samples and TCGA data were provided by the *Recount2* protocol and differential gene expression (DEG) analyses were carried out using the *limma*-*voom* pipeline in the *TCGAbiolinks* R package. Gene ontology (GO) enrichment analysis was performed using the *pathfindR* R package. Mutational and clinical data were acquired from cBioPortal and used to generate Kaplan–Meier survival curves.

**Results:** Of 472 PID-related genes, 151 (32.0%) up-regulated and 88 (18.6%) down-regulated genes were identified across the 29 unified TCGA-GTEx datasets. GO analysis revealed enriched pathways related to complement and coagulation cascades (27/29), systemic lupus erythematosus (25/29), along with Fanconi anemia (22/29) for the DEGs. Top mutated PID-related genes were FAT4 (12.61%), KMT2D (12.30%), and PTEN (10.84%) across all cancer types, with a total of 72 affected PID-related genes.

**Conclusions:** Our integrative approach aids in the elucidation of molecular mechanisms that bridge PIDs to tumour biology.

## Other Allergy/Immunology

### #48 Transition to practice: lessons learned in allergy and immunology training

#### Chloe E. Cyr^1^, Michael M. Cyr^2^, Jaclyn Quirt^2^, Lori Connors^1^

##### ^1^Dalhousie University, Halifax, NS, Canada; ^2^McMaster University, Hamilton, ON, Canada

**Correspondence:** Chloe E. Cyr

*Allergy Asthma Clin Immunol* 2021, **17(Suppl 1):**48

**Background:** There is currently little Canadian data to assess how well traditional time-based residency training programs have prepared residents for careers in Clinical Immunology and Allergy (CIA). This study aims to identify the perceived preparedness of residents in various areas of practice upon the completion of a Canadian CIA residency training program. In addition, this study will provide a baseline for future research comparing Competence by Design (CBD) and traditional time-based residency programs, with respect to transition to practice competencies.

**Methods:** In the summer of 2020, an electronic survey was sent to 2018 and 2019 graduates of Canadian CIA training programs by the Canadian Society of Allergy and Clinical Immunology (CSACI). Several reminder emails were sent and Program Directors were encouraged to share the survey with their former residents.

**Results:** Former residents felt well prepared in most medical expert areas, particularly to treat allergic rhinitis, urticaria/angioedema, venom allergies, and drug allergies. Residents felt less prepared to treat autoimmune diseases, autoinflammatory disorders, inborn errors of immunity, and to conduct various aspects of practice set-up and management. For example, they felt less prepared to hire office staff, set up a research lab, provide virtual care, and set up an office. The majority of these intrinsic competencies were learned through mentorship and on the job after finishing training.

**Conclusions:** Upon completion of training, Canadian CIA residents felt well prepared for many competencies, particularly in medical expert areas. Training programs may wish to focus on various intrinsic competencies, specifically the Leader role, in order to better prepare residents for transition to practice. Academic half-day was not identified as a primary learning centre, suggesting that new teaching strategies may be required.

### #49 Individual therapeutic trials of tiotropium as add-on to inhaled corticosteroids in children with asthma: a clinical experience

#### Zainab Ridha^1,2^, Hanaa Moussa^2^, Anna Smyrnova^2^, Olivier Drouin^2,3,4^, Aniela Pruteanu^3,4^, Sandrine Essouri^3,4^, Francine M. Ducharme^2,3,4^

##### ^1^Université Laval, Quebec City, QC, Canada; ^2^Centre Hospitalier Universitaire Sainte-Justine Research Centre, Montreal, QC, Canada; ^3^General Pediatrics, Department of Pediatrics, Centre Hospitalier Universitaire Sainte-Justine, Montreal, QC, Canada; ^4^ Department of Pediatrics, Faculty of Medicine, Université de Montréal, Sainte-Justine Hospital, Montreal, QC, Canada

**Correspondence:** Zainab Ridha

*Allergy Asthma Clin Immunol* 2021, **17(Suppl 1):**49

**Background:** Tiotropium is a long-acting bronchodilator authorized by *Health Canada* as an adjunct therapy to inhaled corticosteroids in severe asthmatic adults. International and Canadian guidelines recommended its use in severe asthmatic children ≥ 6 or ≥ 12 years, respectively. The objective of this study was to describe the context of use of and clinical experience with tiotropium in Canadian asthmatic children.

**Methods:** This mixed-method case series study collected parents’ and physicians’ independent perceptions of tiotropium’ use. Asthmatic children aged 1 to 17 years were eligible if enrolled in the *Pediatric Asthma Database and Biobank (PADB)* of the Sainte-Justine University Health Centre and received a tiotropium prescription. Clinical data were collected from the PADB electronic records. The parents/children’s and treating physicians’ perceptions were collected independently by electronic surveys inquiring about reasons for initiation, perceived efficacy and perceived safety on 7-point Likert scales, and overall satisfaction.

**Results:** Between May–August 2020, 34 children were included; 11 (32%) were female with a median (range) age of 10.6 (1.4, 17.8) years, with 79% surveys completed by families and 100% by physicians. All patients initiated tiotropium. Physicians’ non-exclusive prescription objectives were to improve lung function (68%), reduce chronic symptoms (65%), exacerbation severity (32%), exacerbation frequency (29%), and/or to replace medications causing side effects (47%). Most parents (89%) and physicians (71%) were conformable with initiating tiotropium. Overall, 93% of parents and 71% of physicians reported improvement in the child’s asthma. Side effects related to tiotropium occurred infrequently. Adding tiotropium generally didn’t affect the parents’ ability to give other prescribed medications. Most parents (93%) and all physicians would try tiotropium again.

**Conclusions:** Pediatric off-label use of tiotropium, mostly prescribed to relieve chronic symptoms, lung impairment, or side effects, was perceived safe and effective in most children. The later two treatable traits, not previously considered as treatment indication, deserve confirmation in prospective pediatric studies.

### #50 Does the REMA score help to differentiate individuals with systemic mastocytosis from those with hereditary alpha-tryptasemia?

#### Maggie Jiang^1^, Gerald Lebovic^2^, Joshua D. Milner^3^, Jonathan J. Lyons^3^, Martina Trinkaus^4^, Lisa Hicks^4^, Anna Nikonova^4^, Peter Vadas^1^

##### ^1^Division of Allergy and Clinical Immunology, St. Michael’s Hospital, University of Toronto, Toronto, ON, Canada; ^2^Applied Health Research Centre, St. Michael’s Hospital, Toronto, ON, Canada; ^3^Laboratory of Allergic Diseases, National Institute of Allergy and Infectious Diseases, National Institutes of Health, Bethesda, MD, USA; ^4^Division of Hematology, Department of Medicine, University of Toronto, Toronto, ON, Canada

**Correspondence:** Maggie Jiang

*Allergy Asthma Clin Immunol* 2021, **17(Suppl 1):**50

**Background:** Systemic mastocytosis (SM) and hereditary alpha-tryptasemia (HαT) can have overlapping presentations of mast cell activation and are difficult to distinguish on clinical grounds. SM diagnosis requires bone marrow or tissue biopsy whereas HαT can be diagnosed with buccal swab for genetic testing [1].

The Spanish Network on Mastocytosis (REMA) score has been validated as a way to predict mast cell clonality and SM using basal serum tryptase levels, clinical symptoms, and sex [2]. This study aims to determine whether REMA scores differ sufficiently between individuals with SM and HαT such as to confidently rule in or out the need for more invasive investigations like bone marrow or tissue biopsy.

**Methods:** A retrospective chart review of 39 patients with SM and 24 patients with HαT was performed in order to calculate individual REMA scores. A two sample Wilcoxon test was conducted in order to assess the difference in median REMA scores between patients with SM and HαT. Subgroup analysis was done within the SM cohort to compare REMA scores based on KIT D816V mutation and SM subtype. Area under the curve was calculated in order to evaluate the discriminatory property of the REMA score.

**Results:** Median REMA score within the SM cohort was 2 (0.50, 4.00) compared to − 1 (− 1.50, 0.00) within the HαT cohort (p < 0.001). REMA scores in patients with SM did not differ based on KIT mutation status. A REMA score cut-off of 0.5 was able to distinguish SM and HαT with a specificity of 83.3% (67%, 96%).

**Conclusions:** This novel comparison of REMA scores in patients with SM and HαT highlights a potential role for calculated REMA score in informing decisions about the need for invasive testing in patients presenting with symptoms of mast cell activation. Larger, comparative studies should be performed before incorporating REMA scoring into routine care.

**References**

1. Carrigan C, Milner J, Lyons J, Vadas P. Usefulness of testing for hereditary alpha tryptasemia in symptomatic patients with elevated tryptase. The Journal of Allergy and Clinical Immunology: In Practice. 2020;8(6):2066–7.
2. Alvarez-Twose I, González-de-Olano D, Sánchez-Muñoz L, Matito A, Jara-Acevedo M, Teodosio C et al. Validation of the REMA Score for Predicting Mast Cell Clonality and Systemic Mastocytosis in Patients with Systemic Mast Cell Activation Symptoms. International Archives of Allergy and Immunology. 2012;157(3):275–80.

### #51 The impact of a structured education session on pediatric atopic dermatitis severity and family quality of life—a retrospective review of new eczema education sessions

#### Shannon Deane^1,2^, Shauna Filuk^1,2^, Jo-Anne St. Vincent^1,2^, Thomas Rawliuk^3^, Allan Becker^1,2,3^, Elinor Simons^1,2,3^

##### ^1^Section of Allergy & Clinical Immunology, Department of Pediatrics & Child Health, University of Manitoba, Winnipeg, MB, Canada; ^2^The Children’s Allergy and Asthma Education Centre, Children’s Hospital, Winnipeg, MB, Canada; ^3^Children’s Hospital Research Institute of Manitoba, Winnipeg, MB, Canada

**Correspondence:** Shannon Deane

*Allergy Asthma Clin Immunol* 2021, **17(Suppl 1):**51

**Background:** Atopic dermatitis (AD) is the most common chronic skin condition of young children and is a chronically relapsing inflammatory skin disease. AD results in significant morbidity and impacts quality of life (QOL). As has been shown in asthma, additional support provided to families in the form of small-group, interactive education sessions improves understanding, treatment adherence and symptoms [1]. An Eczema Education session has been developed to support children and families with AD.

**Methods:** This retrospective review evaluates the effectiveness of Eczema Education sessions. Children up to 5 years of age with moderate-to-severe AD whose parents received Eczema Education were included. Baseline characteristics and AD management behaviours were assessed. Data collected from an AD severity score (SCORAD—SCORing Atopic Dermatitis) and QOL questionnaires (IDQOL—Infants’ Dermatitis Quality of Life and PASECI—Parental Self-Efficacy and Eczema Care Index) were reported before the initial education session and at 3-month follow-up visits.

**Results:** The median age of children with AD was 22.6 (range 5.3–66.6) months; 62% of children were male. The median baseline SCORAD was 52.6 (range 27.4–78.8), which corresponds with moderate-to-severe AD. The median baseline PASECI scores for management of itch, moisturizing and sleep were 7 (range 0–10), 8 (range 0–10), and 7 (range 0–10), respectively. The median baseline IDQOL score was 15 (range 4–26). At the 3-month follow-up, the median SCORAD was 23.6 (range 6.7–56.8). The median change in SCORAD was − 20.7 (range − 45.9 to 6.7) and in IDQOL was -5.5 (range − 19.0 to 2.0), indicating improvement. At least 50% of participants reported an improvement in their ability to manage itch, moisturizing and sleep after only 3 months in the program.

**Conclusions:** This retrospective review demonstrates an improvement in AD and family QOL following Eczema Education sessions, providing evidence that education and additional time and supports for families are worth the effort.

**Reference**

1. Watson WTA, Gillespie C, Thomas N, et al. Small-group, interactive education and the effect on asthma control by children and their families. CMAJ. 2009; 181(5):257–63.

### #52 Cannabis allergy: from edible to inhalation—case report and challenge

#### Ijaz Ogeer^1^, Khadija Brouilliette^1^, Jason Ohayon^1,2^

##### ^1^Hamilton Allergy, Hamilton, ON, Canada; ^2^Department of Pediatrics McMaster University, Hamilton, ON, Canada

**Correspondence:** Ijaz Ogeer

*Allergy Asthma Clin Immunol* 2021, **17(Suppl 1):**52

**Background:** Cannabis use has increased in Canada over the last few years. While the adverse effects of cannabis use has been extensively documented, little has been reported about it’s association with adverse allergic events. Studies have shown clinical presentation of cannabis use varies from mild to life threatening allergic reactions, depending on the route of exposure. Chronic inhalation of cannabis leads to severe manifestations of bronchitis, asthma and worsening pulmonary function. The case below aims to highlight cannabis hypersensitivity and its associated pulmonary sequelae.

**Case Presentation:** A 26 year old female presented with congestion, throat pruritus, and coughing post inhalation of cannabis. Further questioning revealed emesis to edible cannabis with systemic urticaria. Handling of cannabis products also led to contact urticaria. Past medical history was positive for asthma with a drug history of multiple analgesic medications for treatment of chronic pain. Medical marijunana was used as a daily adjunct for pain relief. Skin prick testing was negative to common indoor and outdoor inhaled allergens and was strongly positive to the patient’s sample of medical marijunana, and hemp seeds. Initial pulmonary function testing revealed FEV1 to be 3.15 L (91%) which decreased to 2.78 L (80%) post cannabis inhalation with reversibility post broncho-dilator. The patient was advised to avoid edible cannabis and when possible decrease marijunana inhalation.

**Conclusions:** Previously there were few reported cases of allergic disease associated with cannabis use. Our case confirmed that edible cannabis induced anaphylaxis and allergic asthma can be exacerbated by inhalation of cannabis, generally used for management of chronic pain. Allergic cannabis reactions may increase as cannabis becomes more socially and legally acceptable. Currently, in Canada, there is no approved therapeutic option in suspected cannabis hypersensitivity; strict avoidance remains the recommended treatment. As cannabis becomes increasing medically indicated, awareness of allergic manifestations is needed for appropriate counselling.

**Statement of Consent:** Written informed consent for this case report was obtained from the patient.

### #53 Characteristics of children with eosinophilic esophagitis in British Columbia, Canada since 2012

#### Bei Yuan Zhang^1^, Lianne Soller^2^, Vishal Avinashi^3^, Edmond S. Chan^2^

##### ^1^Faculty of Medicine, University of British Columbia, Vancouver, BC, Canada; ^2^Division of Allergy and Immunology, Department of Pediatrics, BC Children’s Hospital, Vancouver, BC, Canada; ^3^Division of Gastroenterology, Department of Pediatrics, BC Children’s Hospital, Vancouver, BC, Canada

**Correspondence:** Bei Yuan Zhang

*Allergy Asthma Clin Immunol* 2021, **17(Suppl 1):**53

**Background:** Eosinophilic esophagitis (EoE) is a complex, polygenic disorder that has emerged to be a major cause of difficulty swallowing and food impaction. Consequences in children include failure to thrive, impaired nutrition, and esophageal stricturing. Current literature suggests that symptomatology and disease distribution may vary by ethnicity and other demographics.

**Methods:** Since 2012, BC Children’s Hospital has had the only EoE registry in Canada. We reviewed symptoms, atopic history, and treatment for all children (< 18 years) who prospectively consented to our registry between 2012 to 2020.

**Results:** Of 203 patients, 161 (79.3%) were male and 42 (20.7%) were female. 147 (72.4%) identified as white, 45 (22.2%) as South Asian, 6 (2.9%) as East Asian, 2 (1.0%) as black, and 3 (1.5%) as Latin American. On average, whites, when compared to South Asians, were older at their initial visit (11.05 vs. 9.09 years; p = 0.006). The most common atopic history was eczema in South Asians (55.5%) and self-reported food allergy in whites (49.6%). Children presented with vomiting at an earlier mean age of 5.21 years (95% CI 2.93–7.49) compared to dysphagia at 8.47 years (95% CI: 7.00–9.94) and food impaction at 8.64 years (95% CI: 7.02–10.26). Other symptomatology and treatment did not differ between ethnic groups. Trouble swallowing was the most frequently reported symptom (50.2%) followed by vomiting (39.9%). 85.6% of patients were on medical interventions, 58.8% on dietary restrictions, and 48.8% on both. Families of boys were more likely to seek medical treatments compared to girls (88.5% vs 74.3%; p = 0.025).

**Conclusions:** Our EoE registry continues to have a significant proportion of South Asians and relative lack of East Asians. Apart from preferential medical treatment among boys, symptomatology and atopic history did not vary between demographic groups. Genetic and environmental risk factors that could explain the earlier onset of disease in South Asians demand further investigation.

### #54 Successful desensitization for rabies vaccine in a pregnant woman: a case report

#### Shorooq Banjar^1,2^, Salma AlKhammash^3^, Maryse Peterlini^4^, Natacha Tardio^1^, Geneviève Genest^1^

##### ^1^Department of Clinical Immunology and allergy, McGill University, Montreal, QC, Canada; ^2^King Abdulaziz University, Jeddah, Saudi Arabia; ^3^Department of medicine, Immunology and allergy division, King Abdullah Medical City, Mecca, Saudi Arabia; ^4^Department of Pharmacy, McGill University, Montreal, QC, Canada

**Correspondence:** Shorooq Banjar

*Allergy Asthma Clin Immunol* 2021, **17(Suppl 1):**54

**Background:** Rabies vaccination is associated with over two-fold risk of anaphylaxis compared to other vaccines. While post-exposure prophylaxis (PEP) remains the only effective method to prevent this deadly disease, literature on the management of Rabies vaccine hypersensitivity is scant and no standardized desensitization protocols exist. Pregnancy represents a vulnerable state, complicating any necessary desensitization procedure. We report the case of a pregnant woman with suspected anaphylaxis to Imovax who underwent successful vaccine desensitization at the McGill University Health Centre (MUHC).

**Case Presentation:** A 36-year old G5P4 woman was bitten by a bat at 23-weeks gestation. PEP was initiated with the simultaneous administration of Rabies immunoglobulin and Imovax. Within 15 min, she developed anaphylactic shock requiring 6 doses of Epinephrine. Allergy testing could not be performed, but Imovax was thought to be the most likely culprit of her reaction. Medical consensus was to pursue PEP as per Public Health protocol. Three subsequent Imovax doses were successfully given in the intensive care unit, via an intramuscular 5-step vaccine desensitization protocol designed in collaboration with the MUHC pharmacy department. Obstetrics were consulted to ensure fetal monitoring during the procedure, Anesthesia evaluated the airway prior to desensitization and a consultation in Neonatology was obtained in case of premature birth.

**Conclusions:** Rabies is uniformly fatal and the benefit of completing PEP protocol outweighs the risk of anaphylaxis in cases of Rabies vaccine hypersensitivity. In pregnancy, the risk of desensitization is further compounded by maternal physiological changes and the presence of a viable fetus. This case illustrates the necessity of a multidisciplinary approach in designing a desensitization protocol and managing a high-risk patient who requires subsequent doses of a suspected allergen. To our knowledge, this is the first reported case of Rabies vaccine desensitization during pregnancy and underscore the need to develop specific guidelines regarding medication desensitization during gestation.

**Statement of Consent:** Written informed consent for this case report was obtained from the patient.

### #55 Systemic reactions to subcutaneous allergen immunotherapy: cause and effect modelling of real-world experience

#### Adam Aue^1^, Joella Ho^2^, Harold Kim^3, 4^, Samira Jeimy^4^

##### ^1^Faculty of Medicine, University of Ottawa, Ottawa, ON, Canada; ^2^Schulich School of Medicine and Dentistry, Western University, London, ON, Canada; ^3^Department of Medicine, McMaster University, Hamilton, ON, Canada; ^4^Division of Clinical Immunology and Allergy, Department of Medicine, Western University, London, ON, Canada

**Correspondence:** Adam Aue

*Allergy Asthma Clin Immunol* 2021, **17(Suppl 1):**55

**Background:** Subcutaneous immunotherapy (SCIT) is an effective treatment for allergic rhinoconjunctivitis. However, adverse events, including life-threatening systemic reactions, may occur. Multiple studies have described risk factors that predispose patients to systematic reactions to SCIT, however, the identification and management of risk factors varies in clinical practice, with few guidelines to follow. This study identifies risk factors for systemic reactions to SCIT and provides a quality improvement framework with practice-based solutions.

**Methods:** Through retrospective chart review, we describe 10 cases of systemic reactions requiring epinephrine administration, from a hospital-based, Canadian Allergy clinic administering 4134 injections over a 9-month period.

**Results:** The incidence rate of systemic reactions to SCIT requiring epinephrine administration was relatively high, occurring with 1 in 413 injections (0.24%). 8 of the 10 patients had at least one identified risk factor for a systemic reaction. 6 patients had multiple risk factors. Risk factors included omission of pre-medication in the setting of history of large local reactions to injections, strenuous exercise within 2 h before injection, seasonal exacerbation of allergic rhinitis, recent angiotensin converting enzyme inhibitor use, poorly controlled asthma, and errors in route of administration. All reactions occurred with the immunotherapy vial containing the highest allergen extract concentration.

**Conclusions:** This case series highlights the need for improved patient education and pre-administration screening. We suggest several considerations for SCIT administration: (1) provide patients with take-home safety information; (2) screen patients before each injection, including a review of treatment plan adherence and asthma control; (3) consider adjusting the dosing protocol to include slower dose increase of the most concentrated immunotherapy extract, particularly in high risk patients; and (4) apply additional safety measures in patients with multiple risk factors. Our recommendations are summarized in a team based quality improvement framework with practical solutions for implementation in diverse clinical settings.

### #56 Allergy to sex? Nickel, the culprit of post-coitus hypersensitivity

#### Vince Wu^1^, Khadija Brouillette^1, 2^, Sofiya Portuhay^1, 2^, Vaidehi Bhatt^1^, Wardha Wardha^1^, Ijaz A. Ogeer^1^, Saajida S. Hosein^1^, Jason A. Ohayon^1^

##### ^1^Hamilton Allergy, Hamilton, ON, Canada; ^2^McMaster University, Hamilton, ON, Canada

**Correspondence:** Vince Wu

*Allergy Asthma Clin Immunol* 2021, **17(Suppl 1):**56

**Background:** Nickel allergic patients are advised to avoid nickel contact. In cases of systemic dermatitis, these patients are advised to avoid nickel rich foods as well. However, in rare cases, patient avoidance of nickel may be insufficient.

**Case Presentation:** A 26-year-old female was referred for potential allergy to semen. On two occasions, she presented with vulvar burning and swelling 12–18 h post unprotected coitus without concern for immediate allergic reactions during intercourse. Bacterial and fungal infections were ruled out.

Skin prick testing was performed to a sample of her partner’s semen, revealing an irritant reaction, not considered positive from an allergic perspective. Further history identified a delayed-type hypersensitivity to nickel and she had been careful to avoid nickel rich foods and nickel-containing products. She was patch tested to common contact allergens and a sample of her partner’s semen. The patch was removed 48 h post-application and revealed positives to nickel and thimerosal. At 96 h post application, positives were seen to nickel, colophony, and thimerosal. Patch testing was negative to semen at 48 and 96 h.

Her partner was advised to initiate a nickel-free diet for 2 weeks, prior to continuing with unprotected coitus. A month after initiating a nickel-free diet, the burning and swelling post-coitus resolved. However, a month later, after her partner reintroduced nickel into his diet, the vulvar delayed hypersensitivity reaction reoccurred.

**Conclusions:** Patient avoidance of nickel alone was insufficient in the prevention of a vulvar delayed hypersensitivity reaction post-coitus. However, a nickel-free regimen in her partner’s diet prevented delayed hypersensitivity reactions from occurring. In rare cases, nickel may be passed through bodily fluids, resulting in local allergic inflammation in nickel allergic individuals.

**Statement of Consent:** Written informed consent for this case report was obtained from the patient.

### #57 Do we need to rethink the atopic march? An examination of the evidence

#### Jeremy Penn^1^, Michael Aw^2^, Roma Sehmi^1^, Gail Gauvreau^1^, Hermenio Lima^1^

##### ^1^McMaster University, Hamilton, ON, Canada; ^2^University of Ottawa, Ottawa, ON, Canada

**Correspondence:** Jeremy Penn

*Allergy Asthma Clin Immunol* 2021, **17(Suppl 1):**57

**Background:** The atopic march pathway describes the early life progression of allergic diseases from atopic dermatitis (AD), to allergic asthma (AA), and finally to allergic rhinitis (AR). The model enables clinicians to predict future patient disease status. This review aims to assess the true prevalence of the atopic march through published literature including strength of evidence, alternate disease trajectories, and phenotypic importance.

**Methods:** A literature search from appropriate databases (PubMed, MEDLINE, Web of Science) between 1990–2020 was conducted using the terms “atopic march” and yielded 409 results.

**Results:** Our review found that prevalence of the atopic march is likely overstated in the supporting literature. Evidence of trajectories including those who do not develop AA and “skipped” from AD to AR, and a reverse atopic march trajectory (ARàAAàAD) were found in the literature. AD phenotypes were identified to impact progression through the atopic march. Children with food sensitization and AD at 1 year of age are more likely to develop AA at 3 years than those without food sensitization (aRR = 7.04, 95% CI: 4.13, 11.99; aRR = 0.46, 95% CI: 0.11, 1.93, respectively) [1]. At age 7, children with early-onset/persistent AD had an increased risk of AA development (OR = 5.5), while early-onset AD/early-resolving AD cohorts had a lower risk (OR = 1.56), compared to control. The true prevalence of the atopic march–may be closer to 3.1% of atopic individuals [2,3]. Though we do not refute its existence, the atopic march likely represents a smaller proportion of allergic individuals.

**Conclusions:** Evidence to support the presence of the atopic march was found but limited to a small group of atopic patients. Individuals following atopic march pathway comprise a niche subset of the allergic population. Instead, the atopic march–may be one of many temporal pathways for allergic disease progression.

**References**

1. Tran MM, Lefebvre DL, Dharma C, Dai D, Lou WYW, Subbarao P, et al. Predicting the atopic march: Results from the Canadian Healthy Infant Longitudinal Development Study. J Allergy Clin Immunol. 2018;141(2):601–7.e8.
2. Clark H, Granell R, Paternoster L, Curtin JA, Belgrave D, Simpson A, et al. Differential associations of allergic disease genetic variants with developmental profiles of eczema, wheeze and rhinitis. Clin Exp Allergy. 2019;(May):1475–86.
3. Belgrave DCM, Simpson A, Buchan IE, Custovic A. Atopic Dermatitis and Respiratory Allergy: What is the Link. Curr Dermatol Rep. 2015;4(4):221–7.

### #58 Good’s Syndrome: a case of immunodeficiency, thymoma and unusual mucositis

#### Angeliki Barlas, Amin Kanani

##### Clinical Immunology and Allergy, University of British Columbia, Vancouver, BC, Canada

**Correspondence:** Angeliki Barlas

*Allergy Asthma Clin Immunol* 2021, **17(Suppl 1):**58

**Background:** Good’s Syndrome is a rare adult-onset condition often affecting patients between 40 and 70 years of age and is characterized by immunodeficiency and thymoma. Patients with Good’s Syndrome exhibit features of hypogammaglobulinemia, a reduction of peripheral B-cells and CD4+ lymphopenia. These patients are reported to have increased susceptibility to various infections as well as autoimmunity, however, the literature has not identified oral mucositis as a manifestation of Good’s Syndrome.

**Case Presentation:** A 63-year-old female was diagnosed with Good’s Syndrome following a thymectomy in 2013 with a history of recurrent sinopulmonary infections, absent B-cells and significantly low immunoglobulin levels. Prior to the discovery of the thymoma, there was no history of recurrent or invasive viral, bacterial or fungal infections. She was then initiated on monthly intravenous immunoglobulin (IVIG) replacement therapy that resulted in an improvement in the burden of infections. She was well until May 2019, when she began experiencing painful oral mucositis. There was no history of recent chemotherapy or radiation therapy to suggest a secondary cause and oral swabs were negative for herpes. She was evaluated by a dermatologist and oral pathologist and a diagnosis of oral lichenoid mucositis was made. Despite an increase in the IVIG dose, the oral ulcers did not improve. She was advised to use dexamethasone mouthwash for symptom relief.

**Conclusions:** Good’s Syndrome is a rare acquired disease of combined T- and B-cell immunodeficiency with a thymoma and although there have been reported cases of recurrent oral herpetic infections, the literature has not described presentations of oral mucositis as a manifestation of the disease. This case may describe a new clinical profile and provide a better understanding of morbidity for this complex disease.

Consent to publish was obtained from the patient involved in this study.

### #59 Very late-onset familial Mediterranean fever (FMF) associated with isolated IgG deficiency and poor response to conjugated pneumococcal vaccination

#### Selma Lazizi^1^, Michael Fein^2^

##### ^1^Université de Montréal, Montréal, QC, Canada; ^2^Division of Adult Clinical Immunology and Allergy, McGill University, Montréal, QC, Canada

**Correspondence:** Selma Lazizi

*Allergy Asthma Clin Immunol* 2021, **17(Suppl 1):**59

**Background:** Familial Mediterranean fever (FMF) is the most common monogenic autoinflammatory disease characterized by recurrent febrile episodes and serositis. First attacks usually occur during childhood and rarely after 50 years old. We present here the first case to our knowledge of very late-onset FMF associated with isolated IgG deficiency and poor response to conjugated pneumococcal vaccination.

**Case Presentation:** A 77-year-old man of Ashkenazi Jewish descent consulted the emergency department with fever, hypotension, tachycardia, and myalgias. Investigations revealed leukocytosis (WBC 17) and elevated C-reactive protein (54.4 mg/L), however blood and urine cultures were negative. CT-scan of the head, neck, chest and abdomen ruled out infection and neoplasia. His symptoms resolved within days with supportive care and empiric antibiotics. He had experienced similar symptoms twice in the past 5 years without identifiable etiology. The patient had a history of isolated IgG deficiency, one episode of pneumonia and one episode of sinusitis in his lifetime, but no family history of immunodeficiency or autoinflammatory disease. Immunologic evaluation showed low IgG at 4.88 g/L (normal 7.00–15.00), mildly low IgM at 0.44 g/L (normal 0.50–3.00), normal IgA, normal T and B cell enumerations, and low switched memory B cells (2%). He had a good serologic response to tetanus and haemophilus conjugated vaccines, but a poor response to conjugated pneumococcal vaccination (2/14 serologies measured). In light of hypogammaglobulinemia and unexplained febrile attacks, genetic testing was done, revealing heterozygous MEFV pathogenic variant p.Val726Ala (V726A) on exon 10. The patient was started on low-dose colchicine 0.3 mg/day for recurrent gout and has had no FMF attacks since then.

**Conclusions:** FMF is a clinical diagnosis and should be included in the differential diagnosis of unexplained recurrent febrile episodes, regardless of patient’s age. Infections are a well-known trigger of FMF attacks, and patients with humoral immunodeficiencies and FMF may benefit from earlier immunoglobulin replacement therapy.

**Statement of Consent:** Written informed consent for this case report was obtained from the patient.

### #60 Discovery and Validation of a NF-κB related 25-gene transcriptomic signature for prediction of Overall Survival in Neuroblastoma

#### Mehul Gupta^1^, Sunand Kannappan^1^, Luis Murguia-Favela^2^, Aru Narendran^1^

##### ^1^Division of Pediatrics, Section of Pediatric Oncology and Blood and Marrow Transplantation, Alberta Children’s Hospital and University of Calgary, Calgary, AB, Canada; ^2^Division of Pediatrics, Section of Pediatric Hematology and Immunology, Alberta Children’s Hospital and University of Calgary, Calgary, AB, Canada

**Correspondence:** Mehul Gupta

*Allergy Asthma Clin Immunol* 2021, **17(Suppl 1):**60

**Background:** Neuroblastoma is the most common extracranial solid tumor in children. Though the prognosis of low-risk NB is quite favorable, there exists substantial heterogeneity with regards to patient outcomes, making clinical management challenging. However, emerging evidence suggests that immune activation and inflammation may play a crucial role in the progression and metastasis of neuroblastoma. Nuclear factor-kappaB (NF-κB) has been shown to be a central regulator of inflammation, cellular proliferation, and apoptosis in various malignancies. In this study, we identify a NF-κB related prognostic signature for neuroblastoma through the use of an unbiased machine learning approach.

**Methods:** Microarray expression data and matched clinical information was obtained from the TARGET neuroblastoma project (n = 247). Elastic-net regression was performed to select genes associated with overall survival in this cohort. To validate the prognostic gene signature, microarray expression and clinical information for an independent cohort of neuroblastoma patients (n = 266) was obtained from the Gene Expression Omnibus (accession: GSE85047). Association with survival was accessed using univariate cox and log-rank tests, with predictive utility at 5-years being accessed using receiver operator characteristic curves in both cohorts.

**Results:** We identify a 25-gene transcriptomic signature enriched with genes associated with the activation of the NF-κB pathway (p = 0.007). This signature shows significant association with survival in both discovery (log-rank p < 0.001, cox p < 0.001) and validation (log-rank p < 0.001, cox p < 0.001) cohorts. This significant association with survival is also observed in clinically important subpopulations including those based upon MYCN amplification status, age, and INSS stage. Further, the identified signature appears to be significantly predictive of overall survival at 5-years in both the discovery (AUC = 0.9028) and validation (AUC = 0.8529) groups.

**Conclusions:** We generated a prognostic signature for neuroblastoma, enriched for genes associated with NF-κB pathway activity. This data may improve risk stratification of neuroblastoma patients to inform clinical management and identify potential immunologic targets for future therapeutic development.

### #61 Long-term efficacy and safety of dupilumab in adolescents with atopic dermatitis: results from an open-label extension trial (LIBERTY AD PED-OLE)

#### Andrew Blauvelt^1^, Emma Guttman-Yassky^2, 3^, Iftikhar Hussain^4^, Zhen Chen^5^, Ana B. Rossi^6^, Ashish Bansal^5^

##### ^1^Oregon Medical Research Center, Portland, OR, USA; ^2^Icahn School of Medicine at Mount Sinai Medical Center, New York, NY, USA; ^3^Rockefeller University, New York, NY, USA; ^4^Vital Prospects Clinical Research Institute, Tulsa, OK, USA; ^5^Regeneron Pharmaceuticals, Inc., Tarrytown, NY, USA; ^6^Sanofi Genzyme, Cambridge, MA, USA

**Correspondence:** Andrew Blauvelt

*Allergy Asthma Clin Immunol* 2021, **17(Suppl 1):**61

**Background:** We report efficacy and safety data from 299 adolescent patients (≥ 12 to < 18 years) with moderate-to-severe atopic dermatitis (AD) who previously participated in a dupilumab trial (NCT02407756, NCT03054428, or NCT03050151), and subsequently enrolled in an open-label extension (OLE) study (LIBERTY AD PED-OLE, NCT02612454).

**Methods:** In the OLE study, patients either received dupilumab weekly (2 mg/kg [exposure comparable with that in adolescents receiving the approved every 2 weeks (q2w) dupilumab dose regimen in the phase 3 study] or 4 mg/kg) or the fixed dose of 300 mg every 4 weeks. In case of inadequate response in patients receiving the fixed dose, patients were up-titrated to 200 mg/300 mg q2w at Week 16 based on body weight (200/300 mg q2w for patients weighing less than/more than 60 kg, respectively).

**Results:** 105 patients completed up to Week 52 (data cutoff December 15, 2018). At Week 52, 46/106 (43.4%) of patients achieved an Investigator’s Global Assessment (IGA) score of ≤ 1, and 92/106 patients achieved an IGA score of ≤ 2. Absolute change (standard deviation) as measured from the parent study baseline (PSB) to Week 52 of the OLE study was − 28.2 (14.8) for the Eczema Area and Severity Index (EASI, n = 104); and − 11.8 (6.7) for the Children’s Dermatology Life Quality Index (n = 50). 84/104 (80.8%) and 58/104 (55.8%) of patients achieved ≥ 75% and ≥ 90% reduction from baseline in EASI (EASI-75 and EASI-90) relative to PSB. Treatment-emergent adverse events (TEAEs) were reported in 74.2% of patients; 18.1% of patients had a drug-related TEAE. The most common TEAEs were nasopharyngitis (21.1%), AD exacerbation (19.4%), and upper respiratory tract infections (12.4%); 8.7% of patients experienced conjunctivitis. 5 patients reported serious TEAEs, none of which were study drug related.

**Conclusions:** Data from this OLE trial of dupilumab support the long-term efficacy and safety of dupilumab in adolescents with AD.

### #62 Systematic review and network meta-analysis: Corticosteroid formulations for inducing histological remission of eosinophilic oesophagitis

#### Christoher Ma^1, 5^, Brian Feagan^2, 5^, David Claveau^3^, Liette Landry^3^, Véronique Baribeau^4^, Catherine Beauchemin^4, 6^

##### ^1^University of Calgary, Division of Gastroenterology and Hepatology, Departments of Medicine & Community Health Sciences, Calgary, AB, Canada; ^2^Western University, Department of Medicine, London, ON, Canada; ^3^AVIR Pharma Inc., Blainville, QC, Canada; ^4^PeriPharm Inc., Montreal, QC, Canada; ^5^Robarts Clinical Trials, London, ON, Canada; ^6^University of Montreal, Montreal, QC, Canada

**Correspondence:** David Claveau

*Allergy Asthma Clin Immunol* 2021, **17(Suppl 1):**62

**Background:** Several studies support an allergic etiology for eosinophilic oesophagitis (EoE), where a clinical history of allergies is reported in 60–70% of EoE cases. Moreover, EoE responds well to topical corticosteroids that are typically used to treat asthma. Accordingly, off-label treatment options for EoE include fluticasone inhaler content swallowed and budesonide viscous solution (BVS). Importantly, a budesonide orodispersible tablet (BOT) was recently approved for the treatment of EoE in Canada. Here, we aimed to compare the efficacy of BOT with other topical corticosteroid formulations for achieving histological remission in patients with EoE in a network meta-analysis (NMA).

**Methods:** A systematic literature review was performed using MEDLINE and Embase up to August 1, 2019. Eligible studies evaluated adult patients with a diagnosis of EoE treated with a topical corticosteroid in a randomized controlled trial. The outcome of interest was the proportion of patients achieving induction of histological remission defined as a peak oesophageal eosinophil count of < 5 eosinophils/high-power field. Direct comparisons were made using the Mantel–Haenszel method and an NMA was performed using a fixed effects Bayesian framework with Markov Chain Monte Carlo simulations.

**Results:** A total of 6 trials (n = 447 patients) were included in the quantitative summary. The NMA determined that BOT was associated with a higher odds of histological remission compared to BVS (odds ratio [OR] = 4.9 [95% credible interval [CrI] = 1.4, 19.1]), fluticasone (7.4 [1.7, 34.5]), nebulised swallowed budesonide (NSB) (25.0 [2.9, 247.2]) and placebo (387.6 [97.5, 2,275.6]). Similar findings were observed for direct comparisons. Analysis of the ranking of treatment options based on probability of effectiveness found BOT to be most probable followed by BVS, fluticasone, NSB, and placebo, consecutively.

**Conclusions:** The findings of this NMA suggest that BOT is significantly more likely to achieve histological remission in adult patients with EoE compared to BVS, fluticasone, and NSB.

### #63 Laboratory safety of long-term dupilumab treatment for up to 3 years in adults with moderate-to-severe atopic dermatitis (LIBERTY AD OLE)

#### Melinda J. Gooderham^1,2^, Robert Bissonnette^3^, Zhen Chen^4^, Ana B. Rossi^5^, Faisal A. Khokhar^4^

##### ^1^SKiN Centre for Dermatology, Peterborough, ON, Canada; ^2^Queen’s University, Kingston, ON, Canada; ^3^Innovaderm Research, Montreal, QC, Canada; ^4^Regeneron Pharmaceuticals Inc., Tarrytown, NY, USA; ^5^Sanofi Genzyme, Cambridge, MA, USA

**Correspondence:** Melinda J. Gooderham

*Allergy Asthma Clin Immunol* 2021, **17(Suppl 1):**63

**Background:** Long-term use of systemic treatments for atopic dermatitis (AD) typically requires patient safety monitoring for hematologic and/or organ toxicity. Dupilumab treatment has been shown to have favorable safety and sustained efficacy in adults with moderate-to-severe AD. We present laboratory safety data of adults with moderate-to-severe AD treated with dupilumab in the LIBERTY AD open-label extension (OLE) study.

**Methods:** OLE is a phase 3, multicenter trial assessing treatment with subcutaneous dupilumab 300 mg weekly in adults with moderate-to-severe AD who previously participated in controlled dupilumab studies. Data here (cutoff: December 2018) are from patients who received dupilumab for up to 148 weeks.

**Results:** 2,677 patients were treated in the OLE. Mean levels in hematologic parameters remained fairly stable through Week (Wk) 148: mean (standard error [SE]), hemoglobin 1.3 g/L (0.49); platelets − 18.1 × 10^9^/L (2.65); leukocytes -0.3 × 10^9^/L (0.10); eosinophils − 0.2 × 10^9^/L (0.02); and neutrophils − 0.3 × 10^9^/L (0.09). During the OLE, mean platelet levels decreased but remained within the normal range; mean eosinophil levels increased mildly (0.46 × 10^9^/L, Wks8–12) before declining through Wk148, throughout remaining within the normal range.

Mean levels in serum chemistry analyses remained stable through Wk148: mean (SE), potassium 0.1 mmol/L (0.02); blood urea nitrogen 0.02 mmol/L (0.07); creatinine − 0.8 µmol/L (0.53); aspartate aminotransferase 0.7 IU/L (1.24); alanine aminotransferase 2.6 IU/L (0.93); alkaline phosphatase − 0.8 IU/L (0.81); and bilirubin 0.4 µmol/L (0.26). Mean change in lactate dehydrogenase (LDH) at Wk148 was − 43.4 IU/L (5.17). In patients with high baseline LDH levels, 92% shifted to normal LDH levels by Wk148; no patient shifted from normal to low LDH levels. Mean urine pH and specific gravity levels remained within normal ranges throughout the OLE.

**Conclusions:** There were no clinically meaningful adverse changes in mean values for laboratory safety parameters in dupilumab-treated adults with moderate-to-severe AD. These findings suggest that routine laboratory monitoring for hematology/chemistry parameters is not necessary in this population.

### #64 Parental perceptions of drug allergy delabelling following a graded oral challenge

#### Jennifer L. Protudjer^1,2,3^, Michael Golding^1,2^, Tracy Bouwman^4^, Moshe Ben-Shoshan^5^, Elissa M. Abrams^1,4,6^

##### ^1^The University of Manitoba, Winnipeg, MB, Canada; ^2^Children’s Hospital Research Institute of Manitoba, Winnipeg, MB, Canada; ^3^George and Fay Yee Centre for Healthcare Innovation, Winnipeg, MB, Canada; ^4^Meadowood Medical Centre, Winnipeg, MB, Canada; ^5^McGill University, Winnipeg, MB, Canada; ^6^The University of British Columbia, Vancouver, BC, Canada

**Correspondence:** Jennifer L. Protudjer

*Allergy Asthma Clin Immunol* 2021, **17(Suppl 1):**64

**Background:** Most children diagnosed with drug allergy are not deemed allergic after allergy evaluation. Recent studies support the use of oral challenges to de-label antibiotic allergy. Yet, little is known about families’ perceptions of these challenges, or experiences of living with a misdiagnosis, often for many years. As such, we aimed to describe how families with a child previously labelled as “antibiotic allergic,” but who has subsequently been delabelled, perceive the experience of misdiagnosis and subsequent delabelling.

**Methods:** We performed semi-structured interviews with parents whose children had recently completed a graded oral challenge to confirm/refute antibiotic allergy. Interview transcripts were analysed concurrently, but independently, by two investigators, using thematic analysis. This method involved an initial reading of transcripts to identify surface meaning, and a secondary reading to identify latent analysis. Through this two-step process, we developed preliminary coding, which were systematically applied across all data, and from which we subsequently identified themes.

**Results:** A total of 15 parents (14 mothers; 1 mother-father dyad) were interviewed. Children (5/14 [35.7%] boys) were, on average, 5.0 ± 4.5 years, and were first diagnosed in infancy (mean age: 1.8 ± 1.5 years), and commonly at a walk-in clinic (6/14; 42.9%), subsequent to a red, raised rash that appeared within a few days of exposure to a prescribed course of antibiotics. We identified four themes: (1) A red, raised rash results in a quick diagnosis of drug allergy despite lack of testing, (2) sensitive care allays concerns, (3) delabelling brings relief, but also mystery and calls for proper diagnoses, and (4) quick diagnoses are cursory, but manageable through downward comparisons.

**Conclusions:** These findings underscore the importance of a careful physical examination and clinical history of the patient, but also an ongoing dialogue to support families, both of which would ideally begin at the time of initial investigation.

### #65 First-generation H1-antihistamine prescribing in hospitalized patients: a quality improvement measure

#### Sarah K. Lohrenz^1^, Sandy Kassir^2^, Andrea Fong^1^

##### ^1^University of Saskatchewan, Regina, SK, Canada; ^2^Saskatchewan health region, research department, Regina, SK, Canada

**Correspondence:** Sarah K. Lohrenz

*Allergy Asthma Clin Immunol* 2021, **17(Suppl 1):**65

**Background:** First-generation H_1_-antihistamines such as diphenhydramine are commonly utilized in hospital however, these agents are associated with a number of undesirable adverse effects such as central nervous system (CNS) depression, cardiotoxicity and death in overdose. Despite this, prescribers favor first-generation agents, likely due to familiarity and name longevity, over the newer and safer second-generation agents. We aim to establish the proportion of first-generation antihistamines prescribed at our institution over a 1-year period, and to identify the departments in the hospital that may benefit from education around the potential for serious harm of first-generation antihistamines.

**Methods:** We performed a retrospective analysis of all first-generation antihistamine prescriptions at the Regina General Hospital over the past year from January 1, 2019–January 1, 2020 in order to determine the proportion of first-generation antihistamines prescribed, according to admitting service, age group, and pregnancy status.

**Results:** 6,972 prescriptions were written during the study period and 6,692 (96%) were for first-generation antihistamines. 2330 (96%) of patients over the age of 65, 207 (89%) of patients under the age of 18, and 442 (98%) of pregnant women, received first-generation H_1_ antihistamines as opposed to second-generation agents. The largest number of prescriptions for first generation antihistamines was in surgery with a total of 1,902 prescriptions, followed by internal medicine (1,660), obstetrics and gynecology (875), and intensive care (713).

**Conclusions:** We have identified wards at our institution with the highest rates of first generation-antihistamine prescribing with the surgical, obstetrical and medicine wards being the top three. Physicians, pharmacists, and nurses—especially in these identified areas of the hospital—may benefit from education around the potential for serious harm of first-generation antihistamines and the availability of safer alternatives. Additionally, limiting access to first-generation antihistamines in hospital would encourage safer prescribing habits and familiarity with second-generation agents.

## Urticaria/Angioedema

### #66 Presence of autoantibodies in subjects with chronic spontaneous urticaria: a systematic literature review

#### Melanie M. Wong^1^, Paul K. Keith^2^

##### ^1^Michael G. DeGroote School of Medicine, McMaster University, Hamilton, ON, Canada; ^2^Division of Clinical Immunology and Allergy, Department of Medicine, McMaster University, Hamilton, ON, Canada

**Correspondence:** Melanie M. Wong

*Allergy Asthma Clin Immunol* 2021, **17(Suppl 1):**66

**Background:** While the pathogenesis of chronic spontaneous urticaria (CSU) remains unknown, autoantibodies have been found in subjects with CSU. It has been speculated that these autoantibodies cause the condition. Thus, the objective of this review was to investigate the presence of autoantibodies in CSU subjects.

**Methods:** A systematic review was conducted within the PubMed, Medline and CENTRAL databases to identify all studies that assessed the presence of IgG anti-FcεR1α, IgG anti-IgE and anti-TPO antibodies in CSU subjects.

**Results:** 27 papers were included in this review. 14 assessed the presence of IgG anti-FcεR1α antibody in CSU subjects. In five papers that studied CSU subjects with positive-autologous serum skin tests (ASST), 58% had IgG anti-FcεR1α, while in 3 papers that studied CSU subjects with negative-ASSTs, 22.9% had IgG anti-FcεR1α. In nine studies where ASST was not performed, IgG anti-FcεR1α was detected in 43.1% of CSU subjects. In 11 controlled studies, 38.8% of the CSU population had IgG anti-FcεR1α, compared to 6.7% of healthy controls (p < 0.0001). CSU subjects were 6.5 times more likely to have IgG anti-FcεR1α present (p = 0.001). Five studies found IgG anti-IgE antibody in 41.8% of CSU subjects. In four controlled studies, 44% of CSU subjects had this autoantibody present, compared to 15.3% in healthy controls (p = 0.09). CSU subjects were 2.4 times more likely to have IgG anti-IgE present (p = 0.03). In 11 studies, anti-TPO antibody was detected in 17.8% of CSU subjects. In six controlled studies, anti-TPO was found in 16.9% of CSU subjects compared to 5.1% in healthy controls (p = 0.03). CSU subjects were 5.0 times more likely to have anti-TPO present (p = 0.02).

**Conclusions:** Elevated levels of FcεRIa-specific, IgG anti-IgE and anti-TPO autoantibodies were found in CSU subjects compared to healthy controls, which may indicate that autoimmunity may contribute to the pathogenesis of this disease or be an inflammatory marker associated with the condition.

### #67 Don’t forget the poop!” Resolution of chronic spontaneous urticaria with treatment of common stool parasite

#### Khadija N. Brouillette, Collin Terpstra, Jason Ohayon

##### McMaster University, Hamilton, ON, Canada

**Correspondence:** Khadija N. Brouillette

*Allergy Asthma Clin Immunol* 2021, **17(Suppl 1):**67

**Background:** Chronic spontaneous urticaria (CSU) is defined by spontaneous appearance of wheals, angioedema or both for at least 6 weeks. Previous studies have reported an association between parasitic infections and CSU in pediatric patients. While the etiology of CSU is restricted for pediatric patients, there are commonly known etiological factors including thyroid diseases, infections and autoimmune diseases. Currently there is extensive research on these etiological factors, however there is limited literature exploring parasitic infections as a cause for CSU.

**Case Presentation:** A 5 year old female presented with chronic urticaria for the past 4 years. The rash presented as typical urticarial lesions: transient, pruritic skin lesions located ubiquitously. Further examination revealed intermittent angioedema located on the lips and ears. The patient’s urticaria was spontaneous in nature though exacerbated by various: unrelated foods, frigid surfaces, heat, stress, and pressure. Additional questioning precluded common inducible forms of urticarial. Blood work indicated normal thyroid function, negative celiac screening, negative inflammatory markers, and negative autoimmune work up. IgE was elevated at 131 kU/L. Analysis of a stool culture revealed the presence of an amoeba, Dientamoeba fragilis, often felt to be non-clinical relevant in the pediatric gastrointestinal literature. The patient was prescribed Paromomycin for the resolution of the parasitic infection. Subsequently, the patient’s urticarial symptoms reduced in severity by 90%. Patient was advised to perform a repeat stool test.

**Conclusions:** Parasitic infection in relation to CSU have been reported. This case identifies the benefit of further investigational screening in select CSU patients and the eradication for a low toxicity parasitic agent. The gradual increase of parasitic related cases in tropical and non tropical countries would suggest that parasitology should become a part of the routine screening for childhood CSU. Further research is required to determine a causal relationship between parasitic infections and CSU.

**Statement of Consent:** Written informed consent for this case report was obtained from the patient.

### #68 Mild COVID-19 respiratory infection associated with moderate flare of chronic spontaneous urticaria in a 43-year old female long-term care worker on omalizumab

#### Derek Lanoue^1^, Hoang Pham^2^, Jodi Valois^3^, William Yang^3, 4^, Tim Olynych^1, 3^

##### ^1^Department of Medicine, The Ottawa Hospital, The University of Ottawa, Ottawa, ON, Canada; ^2^Department of Medicine, Division of Clinical Immunology and Allergy, McGill University, Montreal, QC, Canada; ^3^Ottawa Allergy Research Corporation, Ottawa, ON, Canada; ^4^Faculty of Medicine, University of Ottawa, Ottawa, ON, Canada

**Correspondence:** Derek Lanoue

*Allergy Asthma Clin Immunol* 2021, **17(Suppl 1):**68

**Background:** Viruses, including COVID-19, are known triggers of urticaria. Omalizumab, an anti-IgE monoclonal antibody, is indicated in chronic spontaneous urticaria (CSU) patients who are refractory to antihistamines. Omalizumab has been found to reduce the duration, frequency, and viral shedding of rhinovirus infections. We hypothesize that CSU patients on omalizumab may have a decreased risk of severe infection with COVID-19. As such, we describe a long-term care worker on omalizumab who experienced a mild COVID-19 infection with CSU flare.

**Case Presentation:** We present a 43-year old Caucasian, non-obese, non-atopic female with a 20-year history of CSU with eosinopenia who was well-controlled on cetirizine 10 mg/day and omalizumab 300 mg SC q4weeks since 2017 with an Urticaria Activity Score 7 (UAS7) of 0 on a background of severe anxiety/depression and active smoking (20-pack year). She had normal lung function and no cardiac comorbidities. On March 30th, 2020, she tested positive for COVID-19 after 5 days of worsening nasal congestion, headache, and fever, while working at a long-term care facility heavily impacted by a COVID-19 outbreak. Her COVID-19 symptoms resolved without intervention after 7-days, which was followed by a moderate flare of her CSU (UAS7 16). Omalizumab was given the week before symptom onset and 14-days after symptom resolution. She did not require a burst of prednisone to control her urticarial flare.

**Conclusions:** Despite her smoking history and intense occupational exposure risk, she only had a mild COVID-19 infection. Additionally, despite her high underlying stress levels, baseline eosinopenia, and acute viral infection, she only had a moderate flare of CSU. This case suggests that targeted anti-IgE therapy may lessen the severity of COVID-19 infection and prevent severe exacerbations of CSU, which is theoretically preferable to more broad immunosuppression with corticosteroids. Larger studies are needed to clarify guidance on withholding or continuing omalizumab in CSU patients with COVID-19.

**Statement of Consent:** Written informed consent for this case report was obtained from the patient.

### #69 Review of C1-INH therapy in angiotensin converting enzyme inhibitor or angiotensin receptor blocker induced angioedema—a Manitoba population analysis

#### Uliana M. Kovaltchouk^1^, Boyang Zhang^1^, Vipul Jain^2^, Chrystyna Kalicinsky^1^

##### ^1^University of Manitoba, Winnipeg, MB, Canada; ^2^McMaster University, Hamilton, ON, Canada

**Correspondence:** Uliana M. Kovaltchouk

*Allergy Asthma Clin Immunol* 2021, **17(Suppl 1):**69

**Background:** Angiotensin Converting Enzyme Inhibitors (ACEi) are a common cause of Emergency Room presentation for angioedema. Although no treatment guidelines exist, C1 esterase inhibitor concentrate (C1-INH) is used on an off label basis for management of ACEI acquired angioedema.

**Methods:** A retrospective chart review, from three academic hospitals in Winnipeg, Manitoba, was conducted examining all patients with ACEI induced angioedema, who received Berinert treatment, between 2010 and 2018.

**Results:** Nine patients, from 3 academic sites, were identified through Allergy Service consultation data and records from Diagnostic Services Manitoba, Canada from 2010–2018. The majority of the patients (n = 7/9) required endotracheal intubation prior to the initiation of Berinert. Overall, approximately 44% of patients (n = 4/9) had resolution of angioedema ranging between 12–13.5 h, with a median time of 12.75 h, and no recurrence. One patient had transient symptom resolution in 14 h, however, recurrence of angioedema required re-intubation. The remainder of patients (n = 5/9), had resolution of angioedema between 22–34 h, with a median time of 22 h.

**Conclusions:** This study adds to the current medical literature, as we have specifically investigated the efficacy of C1-INH concentrate administration in patients who have been intubated for airway protection from ACEi induced angioedema. Our findings suggest that administration of C1-INH concentrate may shorten the time spent in the intensive care unit in a subgroup of patients. Ultimately, further research into characterizing this subgroup of patients needs to be completed.

### #70 CSU and autoimmunity: another lead-gluten enteropathy

#### Ijaz Ogeer^1^, Jason Ohayon^1,2^

##### ^1^Hamilton Allergy, Hamilton, ON, Canada; ^2^Department of Paediatrics, McMaster University, Hamilton, ON, Canada

**Correspondence:** Ijaz Ogeer

*Allergy Asthma Clin Immunol* 2021, **17(Suppl 1):**70

**Background:** Chronic spontaneous urticaria (CSU) is characterized by the appearance of chronic/recurrent wheals, angioedema, or both for at least 6 weeks with reproducible triggers in a subset. While autoimmune disease is generally included in the workup of CSU, Celiac disease (CD) testing is generally overlooked. CD classically presents with gastrointestinal (GI) symptoms, but occasionally presents with cutaneous manifestations alone as can be seen in the case below.

**Case Presentation:** A 29 year old female with CSU presented with a history of recurrent episodes of periorbital angioedema and pruritus of the ear and throat most notably during and after exercise. Skin prick testing revealed a positive reaction to common outdoor inhalant allergens and cat. A diagnosis of exercise induced urticaria possibly aggravated by the ingestion of highly allergenic food prior to exercise was made. She was therefore advised to avoid consumption of the relevant foods before commencing her exercise routine. Despite having adhered to medical advice, the patient required further assessment due to persistent symptoms and new onset nausea. Blood work identified an elevated Thyroid Stimulating Hormone, Anti Thyroid Peroxidase and Anti Tissue Transglutaminase indicating an autoimmune etiology. A subsequent Gastroenterology consult confirmed CD on endoscopic biopsy. At follow up improvement of her urticaria and facial swelling improved after implementation of a gluten free diet.

**Conclusions**: Leznoff et al. originally identified association of CSU patients with the presence of antithyroid antibodies suggesting an autoimmune link [2]. Thyroid testing including autoantibody thyroid evaluation is now common practice in CSU. The above case highlights the benefit of CD screening in mildly GI symptomatic patients with CSU. Evaluation on functional inquiry of GI symptoms with antibody screening will help identify patients where gluten avoidance may be helpful in CSU management.

**References**

1. Bernstein JA, Lang DM, Khan DA, Craig T, Dreyfus D, Hsieh F, et al. The diagnosis and management of acute and chronic urticaria: 2014 update. J Allergy Clin Immunol. 2014;133(5):1270
2. Haussmann J, Sekar A. Chronic urticaria: a cutaneous manifestation of celiac disease. Can J Gastroenterol. 2006;20(4):291–3.

**Statement of Consent:** Written informed consent for this case report was obtained from the patient.

### #71 Salt-dependent aquagenic urticaria: case of a rare physical urticaria

#### Peter Stepaniuk, Amin Kanani

##### University of British Columbia, Vancouver, BC, Canada

**Correspondence:** Peter Stepaniuk

*Allergy Asthma Clin Immunol* 2021, **17(Suppl 1):**71

**Background:** Salt-dependent aquagenic urticaria is a rare physical urticaria which occurs in response to cutaneous exposure to ocean/sea water. There have been less than a dozen reported cases in the literature, and this condition appears to primarily affect young women. Confirmation of diagnosis with provocation testing using a cloth soaked in 3.5% NaCI water solution at room temperature and applied to the patient for 20 min is recommended.

**Case Presentation:** We present a case of a 19-year-old female presenting with an eight-year history of recurrent urticaria after swimming in the ocean. She could tolerate bathing at home and had gone swimming multiple times in freshwater lakes and swimming pools with no adverse reaction. She denied any systemic symptoms suggestive of anaphylaxis. She otherwise had a history of exercise induced urticaria and allergic rhinoconjunctvitis with sensitization to tree and grass pollen. Provocation testing had previously been negative on multiple occasions for cold-induced and aquagenic urticaria when conducted with freshwater. A paper towel was soaked in room temperature ocean water and applied to the patient’s chest for 20 min. The paper towel was removed and 20 minuets after removal, 1–3 mm pruritic wheals, with associated flare developed on her chest where the ocean-soaked paper towel had been. The patient reported improvement of her symptoms with avoidance and use of non-sedating antihistamine therapy.

**Conclusions:** We identified a case of salt-dependent aquagenic urticaria in a young female patient, with convincing history and confirmatory provocation testing. Although extremely rare, salt-dependent aquagenic urticaria is a described clinical entity that should be suspected in patients who develop urticaria after exposure to ocean/sea water. Diagnosis can be confounded by other physical urticarias, such as cold-induced urticaria, cholinergic urticaria, and standard aquagenic urticaria. Provocation testing can confirm the diagnosis and reassuringly, most patients respond to treatment with non-sedating antihistamines.

**Statement of Consent:** Written informed consent for this case report was obtained from the patient.

